# AYURAKSHA, a prophylactic Ayurvedic immunity boosting kit reducing positivity percentage of IgG COVID-19 among frontline Indian Delhi police personnel: A non-randomized controlled intervention trial

**DOI:** 10.3389/fpubh.2022.920126

**Published:** 2022-08-16

**Authors:** Tanuja Nesari, Sujata Kadam, Mahesh Vyas, Vitthal G. Huddar, Pradeep Kumar Prajapati, Manjusha Rajagopala, Anand More, Shri krishna Rajagopala, Santosh Kumar Bhatted, Rama Kant Yadav, Vyasdeva Mahanta, Sisir Kumar Mandal, Raja Ram Mahto, Divya Kajaria, Rahul Sherkhane, Narayan Bavalatti, Pankaj Kundal, Prasanth Dharmarajan, Meera Bhojani, Bhargav Bhide, Shiva Kumar Harti, Arun Kumar Mahapatra, Umesh Tagade, Galib Ruknuddin, Anandaraman Puthanmadam Venkatramana Sharma, Shalini Rai, Shivani Ghildiyal, Pramod R. Yadav, Jonah Sandrepogu, Meena Deogade, Pankaj Pathak, Alka Kapoor, Anil Kumar, Heena Saini, Richa Tripathi

**Affiliations:** ^1^All India Institute of Ayurveda (AIIA), New Delhi, India; ^2^Department of Prasuti and Stri Roga (Obstetrics and Gynaecology), All India Institute of Ayurveda (AIIA), New Delhi, India; ^3^Department of Maulik Siddhant (Fundamental Principles), All India Institute of Ayurveda (AIIA), New Delhi, India; ^4^Department of Kaya Chikitsa (Internal Medicine), All India Institute of Ayurveda (AIIA), New Delhi, India; ^5^Department of Ras Shastra and Bhaishajya Kalpana (Ayurvedic Pharmaceutics), All India Institute of Ayurveda (AIIA), New Delhi, India; ^6^Department of Shalakya Tantra (Eye and ENT), All India Institute of Ayurveda (AIIA), New Delhi, India; ^7^Department of Roga Nidan and Vikriti Vigyan (Pathology), All India Institute of Ayurveda (AIIA), New Delhi, India; ^8^Department of Bala Roga (Pediatrics), All India Institute of Ayurveda (AIIA), New Delhi, India; ^9^Department of Panchkarma (Penta Bio-Purification Methods), All India Institute of Ayurveda (AIIA), New Delhi, India; ^10^Department of Shalya Tantra (Surgery), All India Institute of Ayurveda (AIIA), New Delhi, India; ^11^Department of Shareer Kriya (Physiology), All India Institute of Ayurveda (AIIA), New Delhi, India; ^12^Department of Dravya Guna (Materia Medica and Pharmacology), All India Institute of Ayurveda (AIIA), New Delhi, India; ^13^Department of Swastha Vritta (Preventive and Social Medicine), All India Institute of Ayurveda (AIIA), New Delhi, India; ^14^Hospital - All India Institute of Ayurveda (AIIA), New Delhi, India

**Keywords:** COVID-19, immunity, Ayurveda, quality of life (QOL), hematological parameters, AYURAKSHA kit

## Abstract

**Objective:**

The world continues to face the COVID-19 crisis, and efforts are underway to integrate traditional medicine interventions for its effective management. The study aimed to determine the efficacy of the “AYURAKSHA” kit in terms of post-interventional percentage of COVID-19 IgG positivity, immunity levels, and quality of life (QoL) against COVID-19.

**Method:**

This was a non-randomized controlled, prospective intervention trial, done after the distribution of 80,000 AYURAKSHA kits (constituent of Sanshamani Vati, AYUSH Kadha, and Anu Taila) among Delhi police participants in India. Among 47,827 participants, the trial group (*n* = 101) was evaluated with the positivity percentage of IgG COVID-19 and Immune Status Questionnaire (ISQ) scores as a primary outcome and the WHO Quality of Life Brief Version (QOL BREF) scores along with hematological parameters as a secondary outcome in comparison to the control group (*n* = 71).

**Results:**

The data showed that the percentage of COVID-19 IgG positivity was significantly lower in the trial group (17.5 %) as compared to the control group (39.4 %, *p* = 0.003), indicating the lower risk (55.6%) of COVID-19 infection in the trial group. The decreased incidence (5.05%) and reduced mortality percentage (0.44%) of COVID-19 among Delhi police officers during peak times of the pandemic also corroborate our findings. The ISQ score and WHO-QOL BREF tool analysis showed the improved scores in the trial group when compared with the controls. Furthermore, no dysregulated blood profile and no increase in inflammation markers like C-reactive protein, erythrocyte sedimentation rate, Interleukin-6 (IL-6) were observed in the trial group. However, significantly enhanced (*p* = 0.027) IL-6 levels and random blood sugar levels were found in the control group (*p* = 0.032), compared to a trial group (*p* = 0.165) post-intervention. Importantly, the control group showed more significant (*p* = 0.0001) decline in lymphocyte subsets CD3^+^ (% change = 21.04), CD4^+^ (% change = 20.34) and CD8^+^ (% change = 21.54) levels than in trial group, confirming more severity of COVID-19 infection in the control group.

**Conclusion:**

The AYURAKSHA kit is associated with reduced COVID-19 positivity and with a better quality of life among the trial group. Hence, the study encourages in-depth research and future integration of traditional medicines for the prevention of the COVID-19 pandemic.

**Clinical trial registration:**

http://ctri.nic.in/, identifier: CTRI/2020/05/025171.

## Introduction

Globally, the 21st-century population is facing an unprecedented COVID-19 pandemic caused by the Coronavirus, SARS-CoV-2, leading to a severe painful healthcare crisis for the entire humanity worldwide. Across the board, as of 13th July 2022, there have been 555,446,890 confirmed cases of COVID-19, including 6,353,692 deaths, reported to WHO (1). India is one of the most affected countries in the world (2) and has recorded more than 43,669,850 confirmed cases of COVID-19 with 525,519 deaths since the first wave of COVID-19 pandemic as per the data available on 13th July 2022 (3). COVID-19 is highly contagious due to lack of immunity among the population (4).

Although, several attempts have been made to understand the exact pathogenesis of the disease (5, 6), the inconsistent presentation of symptoms observed in different individuals may be due to the varying factors like individual constitution, diet, lifestyle, and immunity (7). Currently, there is no specific treatment available to counter this highly contagious disease in conventional medicine, the symptomatic management and the empirical line of management are considered as the standard line of care. In such scenario, one of the strategies remains prevention with reduction of pathogen exposure and enhancing an individual's immunity by traditional medicines. Hence, global efforts are being directed to find a specific cure for the disease by developing SARS-CoV-2-specific antivirals and immunomodulators. Preventive medicine is the core objective of Ayurveda by maintaining the health of a healthy individual by following Dinacharya (Daily regimen), Ritucharya (Seasonal regimen), and Consuming Rasayana (Rejuvenating drugs), one can prevent from being affected by the disease in future. The immunity levels and their importance have been studied related to the levels of pro-inflammatory cytokines, cell-mediated immunity, and adaptive immunity in severe COVID-19 cases (8–10). Hence, the modulation of the immune system is a major strategy in the prevention and treatment of COVID-19 through potential therapeutic immuno-modulators (10). The present COVID-19 challenge has brought a refocus on the traditional systems of medicines as one of the prevention strategies in reducing pathogen exposure and enhancing an individual's immunity. Numerous studies on traditional Chinese medicines and various Asian herbs such as *Tinospora cordifolia, Withania somnifera, Andrographis paniculata, Glycerrhiza glabra, Boerhaevia diffusa*, and *Ocimum sanctum* have demonstrated their potent immunomodulatory, anti-inflammatory properties and their applications for the prevention and treatment of COVID-19 in Asian countries (11–13). Similarly, the formulations like Amla tea were found to be effective in shortening the recovery times of symptoms in COVID-19 patients and have shown an ameliorative effect on the severity of clinical signs and CRP levels (14). In the view of COVID-19 outbreak and lockdown in the Capital territory region, New Delhi, India, taking care of police personnel involved in managing discipline, lockdown, preventing violence during lockdowns, helping people with health-related crises, and maintaining the law-and-order rules, became logical to prevent them from the infection by employing the Ayurveda principles in the prevention of disease. In addition, the Ministry of AYUSH, Government of India recommended various self-care guidelines for preventive health measures and immunity-boosting medicines for COVID-19 prophylaxis supported by Ayurvedic literature and scientific publications (15). Therefore, the police personnel (on duty in the Capital territory region, New Delhi, India) during the first wave of COVID-19, were provided with “AYURAKSHA” (meaning which saves and nurtures life) kits by the All India Institute of Ayurveda (AIIA) under the guidance of Ministry of AYUSH, Government of India, as prophylaxis in order to prevent and safeguard them from COVID-19 infection by enhancing their immunity status. The AYURAKSHA kit contained three products such as AYUSH Kadha, Sanshamani Vati (prepared from *Tinospora cordifolia*), and Anu Taila for nasal application. AYUSH Kadha is constituted of four medicinal herbs (Tulsi, Dalchini, Ginger, and Black Pepper), possessing antiviral, anti-microbial, anti-oxidant, Cyto-protective, and anti-inflammatory properties and helps in promoting immunity (16–18). Sanshamani Vati (*Tinospora cordifolia*), commonly named as Guduchi, boosts immunity and acts as an immune modulator (19). Anu Taila (Oil) nourishes all the sensory organs and helps in relieving congestion in the nostrils, chronic sinusitis (20, 21). The rationale behind utilization of the above drugs was as follows: all the formulations and their suggested doses have been used in Ayurveda since ages with well-documented safety aspects and no visible side-effects (22); all of them were recommended by the Ministry of AYUSH, Government of India, for the prevention of health and boosting immunity for COVID-19 prophylaxis (15); the efficiency of all three drugs and their active ingredients were proved by the evidence of the published literature and reported with anti-viral/reduced viral load and immunomodulatory activity (21, 23, 24), none of them had any toxic effect at the prescribed application dose (22); all of them were cost-effective; easy to use; manufactured in reputed Government approved manufacturing unit. The current study was aimed to determine the efficacy of AYURAKSHA kit in terms of post-interventional determination of incidence of COVID-19 infection, immunity levels, quality of life (QoL) against COVID-19, and determining changes in hematological and biochemical parameters among trial group as compared to control group participants.

## Methodology

### Subjects and methods

#### Study design

This was a prospective, prophylactic interventional, non-randomized controlled trial. A non-randomized study design was adopted as randomization was not feasible due to the lethality of the COVID pandemic and hence the convenient sampling was done. It was a public health intervention, done by the All India Institute of Ayurveda with the advice of the Ministry of AYUSH for the prevention of COVID-19 infection by improving the immunity of the Delhi police personnel in the National Capital Territory of Delhi, India. This open-label study was done during the first wave of COVID-19 pandemic and started participant enrolment on 16^th^ May 2020 for 2 months. The study approval was taken from the AIIA-Institutional Research Board (IRB) and the Institutional Ethics committee (IEC) before the commencement of the study which was further registered under the Clinical trials registry-India (CTRI/2020/05/025171). Written informed consent was obtained from all the participants.

### Participants

This study was done in two parts.

#### Part-1

**Figure 1:**
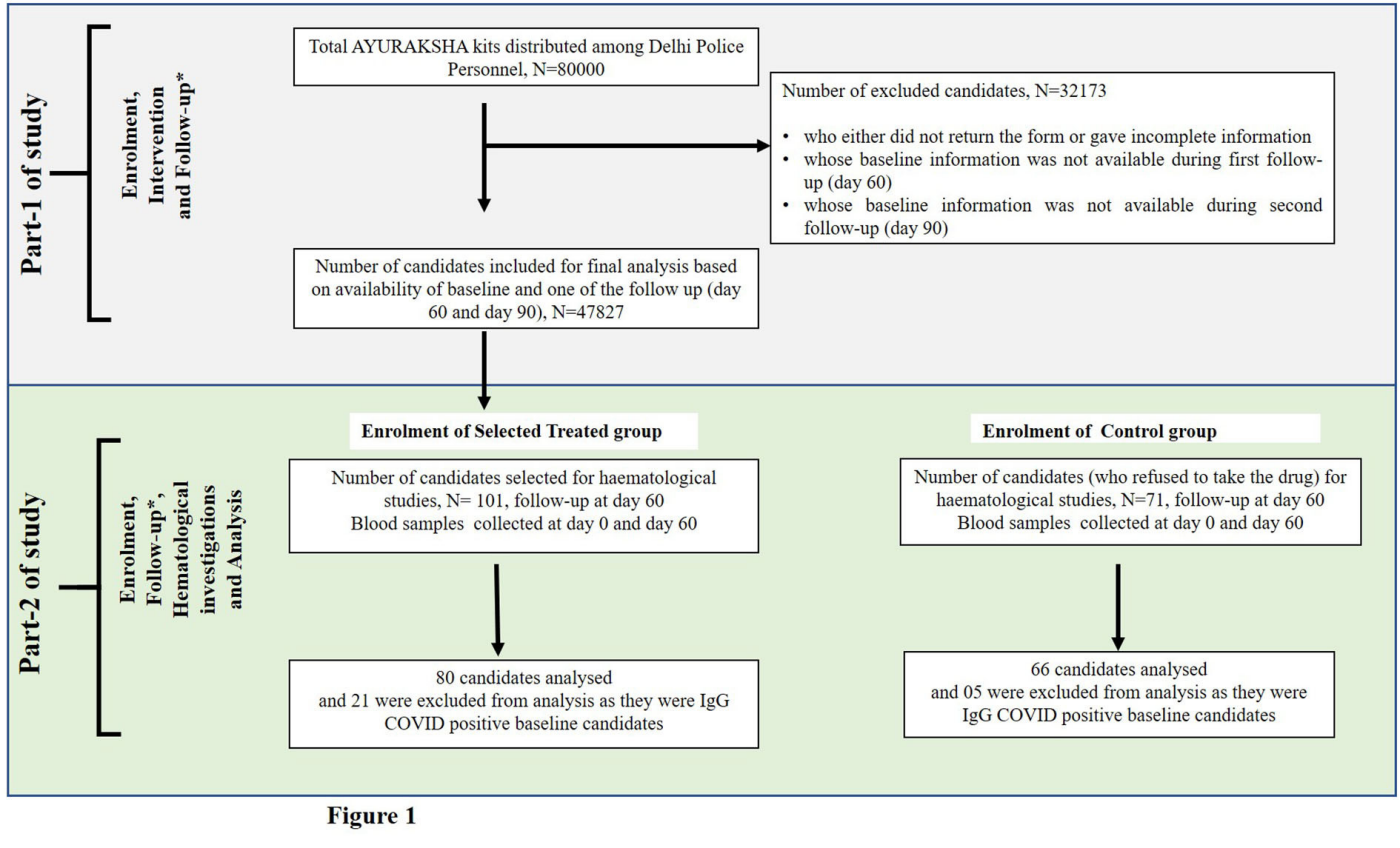
Flow-chart showing the number of candidates included in the non-randomized trial of two groups. ^*^ In part 1 of study, follow-up was done at day 60 and day 90, and in part-2 of study, follow-up was done at day 60.

Part-1 was to distribute AYURAKSHA kit as a public health intervention aiming to provide the prophylactic protection to 80,000 Delhi police personnel scattered in the unit/districts (*n* = 15) of the Capital territory region, New Delhi, India against COVID-19. This was done under the guidelines given by the Ministry of AYUSH. Out of 80,000 participants, the final analysis was done on 47,827 subjects and the details are presented in Figure 1. The data were recorded at baseline (day 0), after intervention at day 60 (first follow-up) and day 90 (second follow-up).

#### Part-2 (main study)

This part of the study was designed as a non-randomized controlled intervention with two parallel groups. The selected *Trial group* participants (from the part-1 study, whose intervention was given with AYURAKSHA kit) and *the Control* group (who refuse to take AYURAKSHA kit) participants were enrolled.

#### Sample size calculation

Since, there was no data available on the spread of COVID-19 infection among the Delhi police personnel, and due to the widespread of COVID 19 infection in Delhi and high exposure of Delhi police personnel, it was assumed that 80% of police personnel would be affected by COVID-19 and the intervention of AYURAKSHA kit for 2 months would reduce this infection by 25–55 percentage points. Assuming the power of the test as 80% and level of significance as 5% and two-tailed test, the calculated sample size was found as 54 in each group. Hence, assuming 20% minimum drop out, it was inflated to 68 in each group. This was the minimum sample size to be taken in each group.

Sample size calculation formula:


$$\begin{array}{l} \text{N}=\;[{\text{Z}}_{1-\alpha /\text{2}}\surd \left(\text{2PQ}\right)\;+\;{\text{Z}}_{\text{1}-\beta }\surd (\text{P1Q1}\\ \;{+\text{P2Q2})]}^{2}/{\left(\text{P1}-\text{P2}\right)}^{\text{2}} \end{array}$$

where P + Q = 1; Q = 1 – P; P = P1 + P2/2; P1: Proportion in the trial group; P2: Proportion in the control group; α: Significance level; 1 – β: Power.

For an extensive study of hematological and biochemical parameters associated with COVID-19 infection, out of 47,827, 101 subjects were enrolled non-randomly as the trial group. Apart from 47,827 subjects, 71 subjects were enrolled as the control group (Figure 1).

*Trial group, n* = 101: AYURAKSHA kit intervention was given for 60 days.

*Control group, n* = 71: These subjects refused to take the kit and hence, no AYURAKSHA kit was given.

During the initial assessment of IgG COVID-19 antibody from the serum of all subjects, we had to exclude 21 subjects from the trial group (out of 101) and 05 (out of 71) subjects from the Control group who were found IgG COVID-19 positive. Thus, the final analysis was performed on 80 candidates in trial group and 66 in the control group who were antibody negative before intervention (baseline or day 0).

The conventional COVID-19 preventive guidelines issued by the Ministry of AYUSH and Ministry of Health and Family Welfare with an illustrative guide on COVID-appropriate behaviors were given to both the groups (15). Also, in both the groups, the blood samples were withdrawn along with the recording of data on all subjects at day 0, at the start of the trial and at day 60 after the treatment (AT).

#### Inclusion criteria for trial and control group

Participants should be

1. Either sex aged 19–60 years,
2. On duty in different units and districts of Delhi state,
3. Agreed to give consent for participation, and
4. Agreed not to take any other prophylactic medicines during the trial period.

#### Exclusion criteria for trial and control group

Participants should not be

1. Suffering with severe respiratory allergies and other co-morbid conditions that may create bias in the outcome of the results,
2. Infected with COVID-19 recently (within 1 month),
3. Positive for IgG COVID-19, and
4. On other prophylactic medications.

### Procedures

The “AYURAKSHA” kits (Immunity enhancer kit) were procured from the Indian Medicine, Pharmaceutical Corporation limited (IMPCL), India, an ISO 9001:2008 and GMP Certified company in 2020 (Batch numbers for **Ayush Kwath-**19-AKC-LDA-063, 19-AKC-LDB-103, 19-AKC-LDA-104, 19-AKC-LDA-065, 19-AKC-LDA-066; **Sanshamani Vati-**43-AVG-LDB-052, 43-AVG-LDB-057, 43-AVG-LDA-426, 43-AVG-LDA-414; **Anu Taila-** 43-ATA-LDB-331; Supplementary Annexure 1). The Ayurveda Prophylactic intervention given (dose for 60 days) included the following: Samshamani Vati (Tablet containing Guduchi, *Tinospora cordifolia*, 500 mg, BD after lunch and dinner) (25), AYUSH Kadha (3 g once a day, decoction of medicated herbs) (26) and Anu Taila (medicated oil for instillation, two drops into each nostril, twice a day) (21, 22). The drug dose was instructed to reduce or stop if any adverse events (e.g., burning chest and stomatitis) were observed during the trial period. During this period, the use of other prophylactic drugs for COVID-19 like Hydroxychloroquine (HCQ) was strictly prohibited.

#### Study tool and data collection

A questionnaire was prepared in consultation with Public Health Foundation of India (PHFI), comprising detailed demographic data, including personal information, Immune Status Questionnaire [validated ISQ (27), questions to assess the symptoms, occurrence, severity and self-assessed health status, part of SF-36 QoL questionnaire, Supplementary Annexure 2], along with WHO-Quality of Life Brief Version (QOL BREF) (28) for the assessment. The questionnaire was handed over and explained by the investigators to the nodal officers of the Police Department who coordinated with the filling of responses at baseline (day 0) and after intervention (AT, day 60) of the study.

#### Laboratory investigations

The status of IgG COVID antibodies in trial and control group candidates was analyzed using ELISafeQ COVID-19 IgG Quantitative ELISA Detection Kit (from Syngene) and COVID positivity was compared. Hematological and biochemical parameters such as CBC (Complete blood count), ESR (Erythrocyte Sedimentation Rate), LFT (Liver function test), Lipid profile, RBS (Random blood sugar), CRP (C-reactive Protein), IL (Interleukin)-2,4, 6,10,12, IgG, IgM, IgA, CD3, absolute CD4^+^, ratio (% CD3^+^/CD45), ratio (% CD3^+^/CD4^+^), absolute CD8^+^, and ratio (%CD3^+^/CD8^+^) were analyzed before and after 60 days in both the trial and control groups.

#### Data management and analysis

The information collected was kept confidential with the investigators only. The excel database of all the participants was created. During the data cleaning, forms with missing or incomplete information were removed (Figure 1). Initially, socio-demographic characteristics of participants using descriptive statistics along with their immunity and health status were evaluated.

#### Compliance of participants

Retention of participants and compliance was assured by random telephone calls and messages to the participants to find out their wellbeing. The compliance was also checked by asking the participants to return the remaining medicines.

#### Data monitoring

The data monitoring committee (DMC) was composed, comprising of staff of AIIA and PHFI (Public Health Foundation of India), independent from the sponsoring body, which trained the investigators regarding the data collected at different time intervals. Further, the investigators coordinated with the nodal officers of the Police Department and provided a brief explanation about the Questionnaires and handed over them, making the cumulative report of the data collected. Data analysis was done by PHFI and AIIA independently from the sponsoring body.

### Outcomes

#### Primary outcomes

- The percentage of susceptible individuals developing an infection (incidence) of COVID-19 was confirmed by measuring IgG antibodies against COVID-19.
- Immune status assessment of an individual was done through ISQ (27).

#### Secondary outcomes

- WHO-QOL BREF, a questionnaire was used for assessing the quality of life (28, 29).
- Hematological and biochemical parameters were studied.
- Adverse events were noted.
- Besides above, as *post-hoc* measures, the comparative analysis of incidence and mortality of COVID-19 was done among Delhi police personnel and the General Delhi population (Supplementary Annexure 3).

### Statistical analysis

All the statistical analysis was done using SPSS version 26 (Chicago, IL, USA). Statistical Analysis was done pre-post using paired *t*-test and their difference with groups tested using an independent *Z*-test. Rate Ratio (Risk Ratio) and its 95% confidence interval is computed to estimate protection due to prophylaxis. Graphical summaries had computed physiologic parameters, health behaviors, and socio-demographic variables.

## Results

All the results of the *part-1* study (*n* = 47,827) including the socio-demographic characteristics, the detailed lifestyle characteristics of participants, health-related parameters measured before treatment (BT), the details of health symptoms during the past 12 months at day 0 (BT), at first (day 60) and second follow-up (day 90) after intervention treatment (AT), the detailed compliance of treatment during the first survey after first (day 60) and second (day 90) follow-up AT, second survey and the detailed immunity and general health status at baseline (day 0), at day 60 after treatment (first follow-up, AT), and at day 90 after treatment (second follow-up, AT) has been depicted in Supplementary Tables S1–S6. During the second survey, the feedback was also taken online via google forms from the participants and it was observed that 22.5% (13,536/60,094) of the participants found it very beneficial; 71.1% (42,825/60,094) found it beneficial and 6.2% (3,707/60,094) responded as “Don't know” (*n* = 60,094; Supplementary Figure S1).

All the results of *part-2* study are as follows:

### The socio-demographic characteristics of study participants

The socio-demographic characteristics of study participants (trial group, *n* = 80, and control group, *n* = 66) are illustrated in Table 1. The mean ± SD of age was found to be 39.39 ± 8.9 years among trial group and 39.47 ± 8.1 among control group, which was further categorized into two sub-categories- ≤ 40 years and >40 years. About 55 and 62.1% of trial and control group participants were of ≤ 40 years of age, and 45 and 37.9% were of >40 years, respectively. The majority of participants (97.5, 90.9%) were males and belonged to the Hindu community (97.5, 98.5%) both in trial and control groups. Education-wise data suggested that among all trial and control group participants, 7.5 and 13.6% were postgraduate, 57.5 and 62.1% were graduates, 27.5 and 18.2% were twelfth, and 7.5 and 6.1% were high school passed. The majority of the police personnel among both the groups were married (98.8 and 100%). Among them, 42.5 and 54.5% were constable, 42.5 and 27.3% were head constable, and 12.5 and 13.6% were inspectors.

**Table 1:** Socio-demographic characteristics of the study participants.

**Characteristics**	**Trial group**	**Control group**
	**(*N* = 80)**	**(*N* = 66)**
**Age in years, Mean** **±** SD	39.39 ± 8.9	39.47 ± 8.1
**Age categories** (%)		
≤ 40 years	44 (55.0)	41 (62.1)
>40 years	36 (45.0)	25 (37.9)
**Gender** (%)		
Male	78 (97.5)	60 (90.9)
Female	02 (2.5)	06 (9.1)
**Religion** (%)		
Hindu	78 (97.5)	65 (98.5)
Other religion	02 (2.5)	01 (1.5)
**Education** (%)		
High school	06 (7.5)	04 (6.1)
Intermediate	22 (27.5)	12 (18.2)
Graduation	46 (57.5)	41 (62.1)
Post-graduation	06 (7.5)	09 (13.6)
**Marital status** (%)		
Unmarried	01 (1.3)	0
Married	79 (98.8)	66 (100)
**Cadre** (%)		
Constable	34 (42.5)	36 (54.5)
HC	34 (42.5)	18 (27.3)
Inspector	10 (12.5)	9 (13.6)
Other	02 (2.5)	3 (4.5)

### The lifestyle characteristics of the study participants

The detailed lifestyle characteristics of participants (trial group, *n* = 80, and control group, *n* = 66) are depicted in Table 2. Most of the police personnel were found to have changed food habit during COVID, 85 and 81.8% of them were found to be homemade food consumers in both trial and control group and only 15 and 18.2% were outside food consumers in both the groups, respectively. It has been observed that only 5.1% were having the habit of consuming smokeless tobacco in trial group and 6.1% in the control group, respectively, 25% were alcohol drinkers in trial group and 24.2% in the control group. About 16.3% of trial and 25.8% of control participants had regular yoga and meditation practice, however, 67.5% of trial group and 45.5% in the control group were irregular in yoga and meditation. Sleep duration at daytime was very less in both trial (0.97%) and the control groups (0.83%) and the mean ± SD of the sleep duration in hours at night was found to be 6.68 ± 1.28 in trial group and 6.59 ± 1.5 in control group, respectively. The body mass index (BMI) was found to be overweight in both the trial group (26.43 ± 4.3) and the control group (26.6 ± 3.6), respectively (Table 2).

**Table 2:** Lifestyle characteristics of the study participants.

**Characteristics**	**Trial group**	**Control group**
***N* (%)/Mean ±SD**	***N* = 80**	***N* = 66**
**Food habit**, ***N*** **(%)**		
Homemade	68 (85.0)	54 (81.8)
Outside food	12 (15.0)	12 (18.2)
Other	0	0
**Chewing tobacco**, ***N*** **(%)**	04 (5.1)	04 (6.1)
**Alcohol drinking**, ***N*** **(%)**	20 (25.0)	16 (24.2)
**Yoga and meditation**, ***N*** **(%)**		
Regular	13 (16.3)	17 (25.8)
Irregular	54 (67.5)	30 (45.5)
Never	13 (16.3)	19 (28.7)
**Day time sleep**, (in h), Median	0	0
Mean **±**SD	0.36 ± 0.97	0.26 ± 0.83
Night time sleep duration in h, Median	7.0	7.0
Mean ± SD	6.68 ± 1.28	6.59 ± 1.5
BMI: Mean ± SD	26.43 ± 4.3	26.6 ±3.6

### Compliance of the treatment

Table 3 showed the detailed compliance with the treatment after the first follow-up (60 days) AT in trial (*n* = 80) groups. During the follow-up AT, it was observed that the total percentage (regularly/irregularly) of compliance response rate of tablets of Samshamani Vati *(T.cordifolia)* were consumed by 91.2% of trial participants. Similarly, AYUSH Kadha and Anu Taila (oil) was consumed by 96.2% of the trial group participants.

**Table 3:** Compliance of the treatment.

**Compliance *N* (%)**	**TT, *N* = 80**
**Tablets**	
Regular	60 (75.0)
Irregular	13 (16.3)
Not taken	07 (8.7)
**Kadha**	
Regular	62 (77.5)
Irregular	15 (18.8)
Not taken	03 (3.7)
**Anu Taila application**	
Regular	62 (77.5)
Irregular	14 (17.5)
Not taken	04 (5.0)

#### Primary outcomes

- Trial group had reduced COVID-19 infection as compared to the control group

As analyzed post intervention, out of 80 candidates in the trial group, 66 remained IgG COVID-19 negative (82.5%) and 14 (17.5%) were found positive. About 40 out of 66 in the control group remained IgG COVID-19 antibody negative (60.6%), but 26 (39.4%) became positive (Table 4). The risks ratio of antibody positive (%) in the trial group vs. control group was found to be 0.444 (0.253–0.779), *p* = 0.003, suggesting 55.6% protection in the trial group as compared to the control group, indicating significant lower risk of COVID-19 infection in the trial group than in control group.

**Table 4:** Impact of prophylactic Intervention on occurrence of COVID-19 infection.

	**Groups**	**Total number of candidates**	**Number of candidates found positive during follow up (day 60)**	**Antibody positive rate (%)**	**Risk ratios (95%CI)**
**1**	**Trial group**	80	14	17.5	0.444 (0.253–0.779)
**2**	**Control group**	66	26	39.4	1.0
				**χ^2^ = 8.72**, ***p*** = 0.003^*^	

* p ≤ 0.05, considered as significant.

b. ISQ analysis

The detailed immunity and general health status of all participants of the trial (*n* = 80) and control (*n* = 66) group at baseline (day 0) and after treatment (day 60, AT) is depicted in Table 5. The data on the qualitative index of general health were measured on a scale of scores between 1 and 10. The data were presented on 2 points, at 0 (bad general health) and 10 (good general health). In Table 5, good immunity status score (6 or more) was described. The data showed a 2.36% increase in mean ± SD of ISQ score from baseline (9.30 ± 1.28) to follow-up AT (9.51 ± 1.08) scores, respectively. However, the values were not found to be significant. In the control group, no change in ISQ score was observed (Table 5). The mean ± SD score of immune functioning was also found to be increased from baseline BT (8.84 ± 1.1) to follow-up AT (9.04 ± 1.2) as compared to their respective controls, however, it was not significant. In addition, the difference in the percentage of a weakened immune system was found to be better in the trial group (5%) participants from baseline BT to AT during follow-up as compared to their respective controls (6.1%).

**Table 5:** Immunity and general health status at baseline and follow-up.

**Immunity and health**	**Trial group** **(*n* = 80)**	**Trial group after intervention follow-up** **(*n* = 80)**	**Control group** **(*n* = 66)**	**Control group follow-up** **(*n* = 66)**
**General health score, Mean ±SD**	8.52 **±** 1.2	8.82 **±** 1.2	8.18 **±** 1.5	8.48 **±** 1.4
**Immunity status**				
**ISQ score, Mean ±SD**	9.30 ± 1.28	9.51 ± 1.08	9.55 ± 0.83	9.56 ± 0.89
**Immune functioning score by participants,** **Mean ±SD**	8.84 **±** 1.1	9.04 ± 1.2	8.24 ± 1.5	8.73 ± 1.3
**Reduced immune function by participants**, ***N*** **(%)**	1 (1.3)	5 (6.3)	0	4 (6.1)

#### Secondary outcomes

a. WHO-QOL BREF

The mean difference (BT-AT) value of domain 1 (physical health) and domain 4 (environment) was found to be significantly increased from baseline (BT, −1.70, *p* = 0.04) to follow-up (AT, −1.67, *p* = 0.02) in trial group, respectively However, no significant difference was obtained in the control group (Table 6).

**Table 6:** WHO QOL BREF domains at baseline and follow-up.

**Immunity and health**	**Trial group** **(*****n*** = **80)**		**TG-TGF,** ***p*****-Value**	**Control group** **(*****n*** = **66)**		**CG-CGF,** ***p*****-Value**
**WHO QOL**	**Baseline** **(TG)**	**Follow-up** **(TGF)**		**Baseline** **(CG)**	**Follow-up** **(CGF)**	
**Domain-1,** **Mean ±SD**	75.57 **±** 1.20	77.27 **±** 1.17	−1.70, ^*^0.04	76.21 ± 1.46	76.32 ± 1.42	−0.11, 0.86
**Domain-2,** **Mean ±SD**	74.85 ± 0.96	75.22 ± 1.31	−0.36, 0.74	74.15 ± 1.73	74.46 ± 1.75	−0.30, 0.18
**Domain-3,** **Mean ±SD**	75.75 ± 1.21	77.62 ± 1.41	−1.87, 0.092	76.00 ± 1.41	77.38 ± 1.52	−1.38, 0.07
**Domain-4,** **Mean ±SD**	73.75 ± 0.89	75.42 ± 1.09	−1.67, ^*^0.026	72.49 ± 1.30	72.98 ± 1.32	−0.49, 0.27

Domain 1, Physical health; Domain 2, Psychological health; Domain 3, Social relationships; Domain 4, Environment.

* p ≤ 0.05, considered as significant.

An increased (non-significant) mean difference (BT-AT) value of domain 2 (−0.36, *p* = 0.74) and domain 3 (−1.87, *p* = 0.09) representing psychological health and social relationships was observed in the trial group while no difference was observed in the control group (Table 6).

b. Reduction in the severity of COVID-19 infection

This study revealed a normal complete blood count (normal Hb, TLC, and platelet count), and no candidate presented with moderate or severe COVID-19 symptoms in the trial group. Notably, as depicted in Table 7, a significant increase in Hb levels was observed in the candidates who took the medicine. The ESR levels got significantly reduced in trial group; however, a higher significant reduction (BT-AT, *p* value) in ESR value was observed with IgG COVID-19 positive ones (4.36, *p* = 0.01) than the ones who stayed negative (2.23, *p* = 0.01) post intervention. CRP level was significantly raised in trial group, but, since it didn't cross the normal range, no severity was observed in any of the subjects. A comparative analysis of BT and AT data for the control group was also done (Supplementary Table S7).

**Table 7:** The blood profile of trial candidates.

**Sr. No**.	**Blood profile**	**IgG COVID-19 antibody (*N*)**	**Paired differences**					***t*-test**	***df***	**Sig. (two tailed, *p*-value)**
			**Mean**	**SD**	**SEM**	**95% confidence interval of the difference**				
						**Lower**	**Upper**			
**1**	**HB_BT-HB_AT**	Positive*-N* = 14	−0.41	0.53	0.14	−0.71	−0.10	−2.89	13.00	0.01^*^
		Negative-*N* = 66	−0.40	0.75	0.09	−0.58	−0.21	−4.30	65.00	0.00^*^
**2**	**TLC_BT - TLC_AT**	Positive-*N* = 14	0.46	2.40	0.64	−0.92	1.85	0.72	13.00	0.48
		Negative-*N* = 66	0.26	1.44	0.18	−0.09	0.61	1.47	65.00	0.15
**3**	**DC_Neutrophils_BT - DC_Neutrophils_AT**	Positive-*N* = 14	0.43	7.56	2.02	−3.94	4.79	0.21	13.00	0.84
		Negative-*N* = 66	−1.47	8.01	0.99	−3.44	0.50	−1.49	65.00	0.14
**4**	**DC_Lymphocytes_BT - DC_Lymphocytes_AT**	Positive-*N* = 14	1.71	6.35	1.70	−1.95	5.38	1.01	13.00	0.33
		Negative-*N* = 66	1.70	6.85	0.84	0.01	3.38	2.01	65.00	0.05^*^
**5**	**DC_Monocyte_BT - DC_Monocyte_AT**	Positive-*N* = 14	−2.57	7.23	1.93	−6.75	1.60	−1.33	13.00	0.21
		Negative-*N* = 66	0.05	5.44	0.67	−1.28	1.39	0.08	65.00	0.94
**6**	**DC_Eosinophils_BT - DC_Eosinophils_AT**	Positive-*N* = 14	−0.21	1.72	0.46	−1.21	0.78	−0.47	13.00	0.65
		Negative-*N* = 66	0.18	2.52	0.31	−0.44	0.80	0.59	65.00	0.56
**7**	**ESR_BT - ESR_AT**	Positive-*N* = 14	4.36	5.50	1.47	1.18	7.53	2.96	13.00	0.01^*^
		Negative-*N* = 66	2.23	6.16	0.76	0.71	3.74	2.94	65.00	0.01^*^
**8**	**PC_BT - PC_AT**	Positive-*N* = 14	−6.71	48.27	12.90	−34.59	21.16	−0.52	13.00	0.61
		Negative-*N* = 66	−17.00	38.71	4.77	−26.52	−7.48	−3.57	65.00	0.00^*^
**9**	**CRP_BT - CRP_AT**	Positive-*N* = 14	−0.61	0.87	0.23	−1.11	−0.11	−2.65	13.00	0.02^*^
		Negative-*N* = 66	−0.54	1.92	0.24	−1.02	−0.07	−2.29	65.00	0.03^*^

* p ≤ 0.05, considered as significant.

c. AYURAKSHA kit reduces the risk of liver abnormalities and maintains the cytokine levels

As depicted in Table 8, the mean difference (BT-AT; *p*-value) showed a significant reduction in total cholesterol (11.14; *p* = 0.01), low density lipoprotein (LDL; 11.45; *p* = 0.00), total bilirubin (0.12; *p* = 0.02), and total protein (0.61; *p* = 0.00) in IgG COVID-19 negative candidates of trial group. The % change (BT-AT; *p* value) in important liver function markers showed more reduction in trial group (SGPT, 13.49; *p* = 0.123 and ALP, 3.06; *p* = 0.308) than in the control group (SGPT, 8.11; *p* = 0.12 and ALP, 1.82; *p* = 0.623). However, the change is not significant (Supplementary Table S8). Strikingly, the % change (BT-AT; *p*-value) showed a reduction in total bilirubin in the trial group (8.91; *p* = 0.08) but a significant elevation in the control group (−12.5; *p* = 0.025; Supplementary Table S8).

**Table 8:** The liver profile of trial candidates.

**Sr. No**.	**Liver profile**	**IgG COVID-19 antibody (*N*)**	**Paired differences**					***t*** **-test**	***df***	**Sig. (two tailed, *p*-value)**
			**Mean**	**SD**	**SEM**	**95% confidence interval of the difference**				
						**Lower**	**Upper**			
**1**	**SGOT_BT-SGOT_AT**	Positive-*N* = 14	3.34	22.76	6.08	−9.80	16.48	0.55	13.00	0.59
		Negative-*N* = 66	5.28	26.84	3.30	−1.32	11.88	1.60	65.00	0.12
**2**	**SGPT_BT-SGPT_AT**	Positive-*N* = 14	9.59	42.27	11.30	−14.81	34.00	0.85	13.00	0.41
		Negative-*N* = 66	6.78	41.91	5.16	−3.52	17.09	1.32	65.00	0.19
**3**	**ALP_BT-ALP_AT**	Positive-*N* = 14	−9.33	39.40	10.53	−32.08	13.42	−0.89	13.00	0.39
		Negative-*N* = 66	5.78	23.60	2.90	−0.03	11.58	1.99	65.00	0.05
**4**	**Albumin_BT-Albumin_AT**	Positive-*N* = 14	0.23	1.07	0.28	−0.39	0.84	0.80	13.00	0.44
		Negative-*N* = 66	−0.01	0.36	0.04	−0.10	0.07	−0.30	65.00	0.76
**5**	**Total Cholestrol_BT - Total Cholestrol_AT**	Positive-*N* = 14	−8.00	20.38	5.45	−19.76	3.77	−1.47	13.00	0.17
		Negative-*N* = 66	11.14	35.55	4.38	2.40	19.88	2.55	65.00	0.01^*^
**6**	**Triglyceride_BT-Triglyceride_AT**	Positive-*N* = 14	−21.91	82.86	22.15	−69.75	25.93	−0.99	13.00	0.34
		Negative-*N* = 66	20.63	104.00	12.80	−4.94	46.20	1.61	65.00	0.11
**7**	**HDL_BT-HDL_AT**	Positive-*N* = 14	13.59	13.17	3.52	5.98	21.19	3.86	13.00	0.00^*^
		Negative-*N* = 66	10.72	17.56	2.16	6.40	15.03	4.96	65.00	0.00^*^
**8**	**LDL_BT-LDL_AT**	Positive-*N* = 14	−5.19	28.48	7.61	−21.63	11.26	−0.68	13.00	0.51
		Negative-*N* = 66	11.45	27.58	3.40	4.67	18.23	3.37	65.00	0.00^*^
**9**	**Bilirubin_BT-Bilirubin_AT**	Positive-*N* = 14	−0.06	0.68	0.18	−0.45	0.33	−0.33	13.00	0.75
		Negative-*N* = 66	0.12	0.38	0.05	0.02	0.21	2.50	65.00	0.02^*^
**10**	**Bilirubin Conjugated_BT-Bilirubin Conjugated_AT**	Positive-*N* = 14	−0.05	0.13	0.04	−0.13	0.02	−1.47	13.00	0.17
		Negative-*N* = 66	0.03	0.30	0.04	−0.04	0.10	0.80	65.00	0.43
**11**	**Bilirubin Unconjugated_BT-Bilirubin**	Positive-*N* = 14	−0.03	0.64	0.17	−0.40	0.34	−0.20	13.00	0.85
	**Unconjugated_AT**	Negative-*N* = 66	0.14	0.40	0.05	0.04	0.24	2.77	65.00	0.01^*^
**12**	**Total Protein_BT-Total Protein_AT**	Positive-*N* = 14	0.28	0.75	0.20	−0.15	0.71	1.39	13.00	0.19
		Negative-*N* = 66	0.61	0.81	0.10	0.41	0.81	6.15	65.00	0.00^*^

BT, before treatment; AT, after treatment.

* p ≤ 0.05, considered as significant.

**Figure 2:**
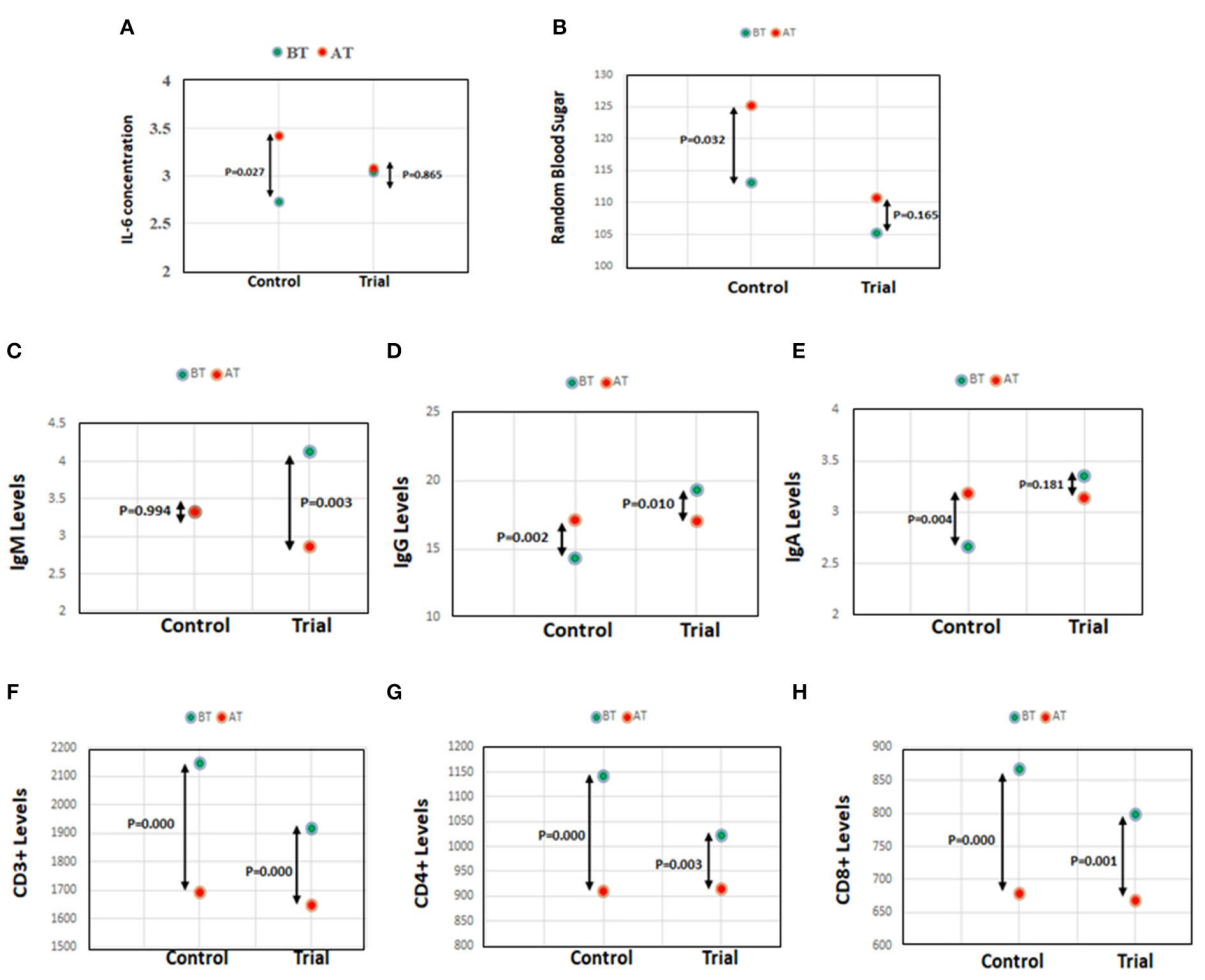
**(A)** IL-6 concentration in control and trial group. **(B)** Random blood sugar levels in control and trial group. **(C–E)** Antibody IgM, IgG, and IgA levels in control and trial group. **(F–H)** Lymphocyte subset, CD3^+^, CD4^+^, and CD8^+^ levels in control and trial group.

Further, no significant change in cytokine levels (IL-2, IL-4, IL-6, IL-10, IL-12, and GI) in the subjects of the trial group (Table 9) was observed. However, IL-6, the most prevalent cytokine in COVID-19 infection, associated with enhanced inflammation and more predictive of death (4, 30) was observed (Figure 2A) to be significantly enhanced (% change BT-AT; *p*-value) in the control group (-25.27; *p* = 0.027) than in trial group (-0.04; *p* = 0.865).

**Table 9:** The cytokine levels in trial candidates.

**Sr. No**.	**Cytokines**	**IgG COVID-19 antibody (*N*)**	**Paired differences**					***t*** **-test**	***df***	**Sig. (two tailed, *p*-value)**
			**Mean**	**SD**	**SEM**	**95% confidence interval of the difference**				
						**Lower**	**Upper**			
**1**	**IL6_BT-IL6_AT**	Positive-*N* = 14	−0.43	2.48	0.66	−1.86	1.00	−0.65	13.00	0.53
		Negative-*N* = 66	0.04	1.96	0.24	−0.44	0.53	0.18	65.00	0.86
**2**	**IL2_BT-IL2_AT**	Positive-*N* = 14	0.38	1.28	0.34	−0.36	1.11	1.10	13.00	0.29
		Negative-*N* = 66	−0.28	2.14	0.26	−0.81	0.24	−1.08	65.00	0.29
**3**	**IL4_BT-IL4_AT**	Positive-*N* = 14	0.36	1.79	0.48	−0.68	1.39	0.74	13.00	0.47
		Negative-*N* = 66	−0.56	5.13	0.63	−1.82	0.70	−0.89	65.00	0.38
**4**	**IL10_BT-IL10_AT**	Positive-*N* = 14	0.11	5.35	1.43	−2.98	3.20	0.08	13.00	0.94
		Negative-*N* = 66	0.70	4.44	0.55	−0.39	1.79	1.28	65.00	0.21
**5**	**GI_BT-GI-AT**	Positive-*N* = 14	−0.01	2.04	0.55	−1.19	1.17	−0.01	13.00	0.99
		Negative-*N* = 66	0.58	3.44	0.42	−0.26	1.43	1.38	65.00	0.17
**6**	**IL12_BT-IL12_AT**	Positive-*N* = 14	−0.26	0.72	0.19	−0.67	0.16	−1.33	13.00	0.21
		Negative-*N* = 66	−0.20	1.30	0.16	−0.52	0.12	−1.24	65.00	0.22

d. AYURAKSHA kit maintained random blood sugar (RBS) levels in trial group

As observed in our study, AYURAKSHA kit maintained the normal blood glucose levels in trial group irrespective of IgG COVID-19 positivity. Interestingly, the RBS level (% change BT-AT; *p*-value) was found to be significantly elevated in the control group (−10.61; *p* = 0.032), while a non-significant change was observed in the trial group (−5.12, *p* = 0.165) post intervention (Figure 2B).

e. Ayurveda-based drug reduces the chance of infection in trial group

As shown in Table 10, a significant decrease (mean difference BT-AT, *p*-value) in total IgM level was observed in IgG COVID-19 positive (2.90; *p* = 0.02) and negative candidates (0.93; *p* = 0.04) of trial group. A reduced level of total IgG (1.74; *p* = 0.04) was also observed in IgG COVID-19 negative candidates of trial group. Interestingly, when the trial group was compared with control group post intervention (% change BT-AT; *p*-value), a significant decrease in the IgM level (Figure 2C) was observed in trial group (30.75; *p* = 0.003) while a significant increase in IgG level (−19.7; *p* = 0.002; Figure 2D) and IgA level (−19.47; *p* = 0.004, Figure 2E) was seen in the control group.

**Table 10:** The antibody levels in trial candidates.

**Sr. No**.	**Antibody type**	**IgG COVID-19 antibody (*N*)**	**Paired differences**					***t*** **-test**	***df***	**Sig. (two tailed, *p*-value)**
			**Mean**	**SD**	**SEM**	**95% confidence interval of the difference**				
						**Lower**	**Upper**			
**1**	**IgM BT-IgM_AT**	Positive-*N* = 14	2.90	4.01	1.07	0.58	5.21	2.70	13.00	0.02^*^
		Negative-*N* = 66	0.93	3.63	0.45	0.03	1.82	2.07	65.00	0.04^*^
**2**	**IgG_BT-IgG_AT**	Positive-*N* = 14	3.63	8.43	2.25	−1.24	8.50	1.61	13.00	0.13
		Negative-*N* = 66	1.74	6.70	0.82	0.10	3.39	2.11	65.00	0.04^*^
**3**	**IgA_BT-IgA_AT**	Positive-*N* = 14	0.18	1.26	0.34	−0.55	0.91	0.54	13.00	0.60
		Negative-*N* = 66	0.23	1.51	0.19	−0.14	0.60	1.23	65.00	0.22

* p ≤ 0.05, considered as significant.

f. AYURAKSHA kit-maintained lymphocyte subset levels in trial group

As shown in Figures 2F–H, the control group showed a more significant decline (% change BT-AT; *p*-value) in lymphocyte subsets CD3^+^ (21.04, *p* = 0.000), CD4^+^ (20.34, *p* = 0.000), and CD8^+^ (21.54, *p* = 0.000) levels than in trial group (CD3^+^ 14.03; *p* = 0.000, CD4^+^ 10.61; *p* = 0.003, CD8^+^ 16.15; *p* = 0.001) post intervention. Notably, as evident from Table 11, the IgG COVID-19 positive trial group candidates showed more reduced (mean difference BT-AT, *p-*value) lymphocyte subsets CD3^+^ (640, *p* = 0.00), CD4^+^ (315.14, *p* = 0.00), and CD8^+^ (265.14, *p* = 0.00) levels than antibody negative candidates in trial group (CD3^+^ 190.61; *p* = 0.01, CD4^+^ 64.65; *p* = 0.08, CD8^+^ 99.97; *p* = 0.02).

**Table 11:** The lymphocyte subsets in trial candidates.

**Sr. No**.	**Lymphocyte subsets**	**IgG COVID-19 antibody (*N*)**	**Paired differences**					***t*** **-test**	***df***	**Sig. (two tailed, *p*-value)**
			**Mean**	**SD**	**SEM**	**95% confidence interval of the difference**				
						**Lower**	**Upper**			
**1**	**CD3_BT-CD3_AT (T-Lymphocytes)**	Positive-*N* = 14	640.00	601.54	160.77	292.68	987.32	3.98	13.00	0.00^*^
		Negative-*N* = 66	190.61	532.16	65.51	59.78	321.43	2.91	65.00	0.01^*^
**2**	**R(%CD3/CD45)_BT-R(%CD3/CD45)_AT**	Positive-*N* = 14	2.89	6.51	1.74	−0.87	6.64	1.66	13.00	0.12
	**(T-Cells)**	Negative-*N* = 66	1.43	4.42	0.54	0.35	2.52	2.63	65.00	0.01^*^
**3**	**Absol_CD4_BT-Absol_CD4_AT**	Positive-*N* = 14	315.14	334.10	89.29	122.24	508.04	3.53	13.00	0.00^*^
	**(T-Helper cells)**	Negative-*N* = 66	64.65	296.68	36.52	−8.28	137.58	1.77	65.00	0.08
**4**	**R(%CD3/CD4)_BT-R(%CD3/CD4)_AT**	Positive -*N* = 14	−0.26	7.03	1.88	−4.31	3.80	−0.14	13.00	0.89
	**(T-Helper cells)**	Negative-*N* = 66	−1.29	5.30	0.65	−2.60	0.01	−1.98	65.00	0.05
**5**	**Absol_CD8_BT-Absol_CD8_AT**	Positive-*N* = 14	265.14	247.05	66.03	122.50	407.78	4.02	13.00	0.00^*^
	**(T-Suppressor cells)**	Negative-*N* = 66	99.97	343.68	42.30	15.48	184.46	2.36	65.00	0.02^*^
**6**	**R(%CD3/CD8)_BT-R(%CD3/CD8)_AT**	Positive-*N* = 14	1.64	3.84	1.03	−0.58	3.86	1.59	13.00	0.14
	**(T-Suppresor cells)**	Negative-*N* = 66	1.40	10.32	1.27	−1.13	3.94	1.11	65.00	0.27
**7**	**R(%CD4/CD8)_BT-R(%CD4/CD8)_AT**	Positive-*N* = 14	−0.08	0.29	0.08	−0.24	0.09	−0.97	13.00	0.35
	**(T-Suppresor cells)**	Negative-*N* = 66	−0.05	0.55	0.07	−0.18	0.09	−0.70	65.00	0.49

* p ≤ 0.05, considered as significant.

g. No adverse events (AE) were noticed by any of the participants in the trial group.

## Discussion

COVID-19 has been the most unpleasant experience for the entire world and a myriad of studies are underway to test a large number of modern and traditional medicines against this virus.

The traditional systems of medicine like Ayurveda promote health through its prophylactic and preventive capabilities and can enhance body immunity in the population to combat the disease. Hence, preventive interventions, including both pharmacological (including Rasayanas and herbal Kadha) (31) and non-pharmacological (Practice of dinacharya) strategies (32) described in Ayurveda must be taken up to combat the COVID-19 pandemic. There are many medicinal herbs or formulations like Ashwagandha (*Withania sominifera*), Tulsi (*Ocimum basilicum* L) (33), curcumin (34), green tea (35), etc., which have immunomodulatory, immune boosting, and anti-viral role. They are reported to inhibit transcription factor 2 (ATF-2), Th17-related cytokines, IL-17A and Th2-related cytokines, including IL-5, IL-13, and IL-6, and increase the secretions of IL-10, INF-γ, etc. (4, 36, 37).

In India, the traditional medicine-based research and development is done under the overarching regulatory body of the Ministry of Ayurveda, Yoga, and Naturopathy, Unani, Siddha and Homeopathy (AYUSH), a federal government organization (http://AYUSH.gov.in/). At the times, when the world was clueless for any treatment, the Ayurveda-based prophylactic intervention (AYURAKSHA kit) was given as a public health measure to the Delhi police participants by AIIA under the guidance of the Ministry of AYUSH, Government of India, in order to improve immunity status, maintain optimum health and to combat with COVID-19 disease during the first wave of COVID-19 in India. The AYURAKSHA kit contains the AYUSH Kwath, Sanshamani Vati, and Anu Taila, which are briefly discussed below for their potential role in fighting infections and immunomodulatory nature.

### AYUSH Kwath

It comprises four medicinal herbs (Tulsi/Holy Basil/*Ocimum sanctum*, Dalchini/Cinnamon/*Cinnamomum zeylanicum*, Sunthi/Ginger/*Zingiber officinale*, and Marich/Black Pepper/*Piper nigrum*), with the formulation composition in the ratio of 4:2:2:1 (26, 38). It promotes immunity and relieves symptoms associated with viral infections due to its immune-modulatory, antiviral, anti-oxidant, anti-inflammatory, anti-platelet, anti-atherosclerotic, hepato-protective, and reno-protective properties (16, 23). Previous publications have demonstrated that all the medicinal constituents of AYUSH Kwath – tulsi (39), cinnamon (40), ginger (41–43), and black pepper (44, 45) are safe to use and have no toxic/genotoxic effects if used as recommended. However, studies have shown that the prolonged use of a few of its constituents, cinnamon (40) and black pepper (44), may have undesirable effects like increased lungs, spleen weight, and oxidative stress.

### *Sanshamani vati* (*Tinospora cordifolia*)

This Ayurvedic herbal formulation is used as a *Rasayana* for all types of fevers. *Tinospora cordifolia* (Thunb.) Miers (TC), commonly named Guduchi, belongs to the family Menispermaceae. It plays a crucial immunomodulatory role either by promoting the phagocytic activity of macrophages or by activating the cytotoxic T cells and B cell differentiation (19, 46) along with hypoglycaemic, antioxidant, anti-hyperglycaemic, antiallergic, anti-inflammatory, and hypogycemic properties (47, 48). Recently, it has been reported for the reversal of the phenotype of the SARS-CoV-2 disease in humanized Zebrafish (24). Previous reports have concluded that *T.cordifolia* (49–52) has no toxic effects.

### Anu Taila (Oil)

Various pharmacological agents, including intra-nasal delivery of TLR2/6 agonist, are studied to prevent the entry of viruses and control the infection (24, 53–55). Ayurveda-based Anu Taila (Oil) derived from several important medicinal plants nourishes all the sensory organs, including nose, and helps in relieving congestion in the nostrils, chronic sinusitis (20, 21), and controlling the pro-inflammatory cytokines (56). Various authors showed a reduced viral load in the lungs after the prophylactic nasal instillation of Anu Taila (21, 22, 24, 57). No toxic effects have been reported using Anu taila (22).

In this study, the efficacy of AYURAKSHA kit was evaluated with the inclusion of two groups, the trial group (to whom the AYURAKSHA Kit was given) and the control group (no treatment was given) from Delhi police and the IgG COVID-19 positivity, immune status, QoL, and hematological parameters were compared. Further, the baseline data analysis of all participants included socio-demographic, lifestyle, immunity, health-related characteristics, and quality of life (QoL) which showed that the enrolled individuals in both the groups have maintained a healthy lifestyle (including food, alcohol, and tobacco chewing habits).

Notably, in an online survey taken among Delhi Police Personnel, the total percentage of compliance response rate of AYURAKSHA medicine was noticed and the results showed that a total of 91.2% participants have taken Sanshamani vati (*Tinospora cordifolia*) tablets and 96.2% have taken Kadha and Anu taila (Table 3). This showed that if 100% participants have taken the AYURAKSHA kit, the protection percentage (55.6%) from COVID-19 may have slightly increased among the trial group. In addition, these results validate the preliminary compliance of part-1 study (Supplementary Table S5) and confirm the acceptance and belief in traditional Ayurvedic medicines among Indian Delhi police (total of 93.6%, Supplementary Figure S1) to enhance their body immunity.

The most striking result of this study is displayed in Table 4 showing that the candidates in trial group were at lower risk of COVID-19 infection (17.5%) than the control ones (39.4%) when analyzed for IgG COVID-19 positivity during follow-up. This data also confirms the role of AYURAKSHA kit in decreasing the incidence and mortality of COVID-19 among Delhi police officers as compared to the general population of Delhi (Supplementary Figures S2a–c, S3a,b). According to a health bulletin released by the Delhi government, the peak of the first wave of COVID-19 was observed in Delhi on 24th June 2020 when the cases in the general population were high (*n* = 3,788 cases in the last 24 h taking the total number of cases to 70,390 and 64 deaths taking the total number of deaths to 2,365). On the same date, Delhi police had reported a clear declined trendline in incidence and mortality of COVID-19 cases (*n* = 33) in spite of high risk of infection due to high exposure (Supplementary Annexure 3, Supplementary Figures S2a–c, S3a,b). Mortality among Delhi Police was found to be 0.44% as compared to 0.95% in the general population (Source: https://www.dnaindia.com/health/report-50-of-COVID-19-deaths-in-age-group-of-above-60-years-68-men-2835908; Supplementary Figure S3b, Supplementary Annexures 2, 3). Importantly, Delhi Police recorded less incidence and mortality as compared to Karnataka, Kolkata, and Mumbai Police (Supplementary Figure S3c), possibly due to the benefits of AYURAKSHA kits (distributed to Delhi police) in preventing the infection of COVID-19.

In order to measure the immunity levels, the validated tool published under the name ISQ (27) was used. This intervention has shown to improve 2.36% ISQ scores as compared to baseline and follow-up after the intervention period, respectively (Table 5).

Quality of life (QoL) refers to the multifaceted concept which includes the four domains of physical health, psychological health, social relationships, and environment of a person (58). The WHO-QOL BREF instrument was developed to measure the above four domains of QoL, through a set of 26 items (28, 29) and can be used across different Nations. Based on this tool, our study has shown a significantly improved QoL of trial group in domain 1 and domain 4 (physical health and environment domain) who took the AYURAKSHA kit as assessed after the intervention periods (Table 6).

Further, the hematological and biochemical parameters which either acted as the biomarker for COVID-19 infection or defined the severity of the disease were analyzed in this study. As per reports, several blood parameters including an elevated CRP, ESR levels, or reduced lymphocytes (59–61) are associated with the severity of COVID-19 infection (61–63). However, no dysregulated blood profile and no increase in inflammation marker were observed in trial group irrespective of IgG COVID-19 antibody status. IgM antibodies are found to be raised in immunological disorders, autoimmune, and acquired infectious diseases (64) as they are produced as a body's first response to an infection and decline after the production of IgG. Interestingly, a reduced level of total IgM, IgG, and IgA was observed in trial group which signifies the decreased incidence of infection with intervention. A plethora of reports have suggested that the liver impairment had been the emerging concern with COVID-19 infection due to direct effect by the virus, immune-mediated inflammation, or drug-induced toxicity (65, 66). Since the use of drugs like lopanivir and ritonavir was associated with severe liver damage in critical COVID-19 patients (67), it was of keen interest to observe the effects of Ayurveda-based drugs on liver abnormalities. Interestingly, no liver-associated risk in trial group was observed in our study. Serum levels of liver test markers like total bilirubin, serum glutamic pyruvic transaminase (SGPT), serum glutamic oxaloacetic transaminase (SGOT), and alkaline phosphatase (ALP) were recorded higher in severe patients with COVID-19 infection (67–69). Our study showed a more reduction in SGPT and ALP levels in trial group than in the control group. Also, a reduction in total bilirubin was seen in trial group, but a significant elevation in the control group (Supplementary Table S8).

Further, Costela-Ruiz et al. (70) and Huang et al. (8) have reported the hyperproduction of cytokines, such as IL-1, IL-6, IL-12, IFN-γ, and TNF-α, preferentially targeting lung tissue, leading to worsening prognosis of COVID-19 infected patients. Strikingly, no significant change in cytokine levels (IL-2, IL-4, IL-6, IL-10, IL-12, and GI) was observed in the trial group. Nevertheless, IL-6 levels, an important cytokine associated with cytokine storm (4) in critically ill COVID patients were also maintained in trial group while there was a significant elevation in the control group. Burgeoning reports have suggested the significance of lymphocyte subsets for the diagnosis and prognosis of COVID-19 infection. Jiang et al. and others have shown a decreased T-cell subset count mainly, CD3^+^, CD4^+^, and CD8^+^ which can be used as diagnostic markers for COVID-19 and are associated with patient severity (71–73). Importantly, the decreased CD3^+^, CD4^+^, and CD8^+^ levels were found to be more significant in the control group than in trial group when analyzed in our study. This implies that the AYURAKSHA kit maintained the cytokine levels and lymphocyte subset levels in the trial group more efficiently than in the control group, hence, highlighted the role of AYURAKSHA Kit as immunity booster during the peak days of Corona pandemic.

**Figure 3:**
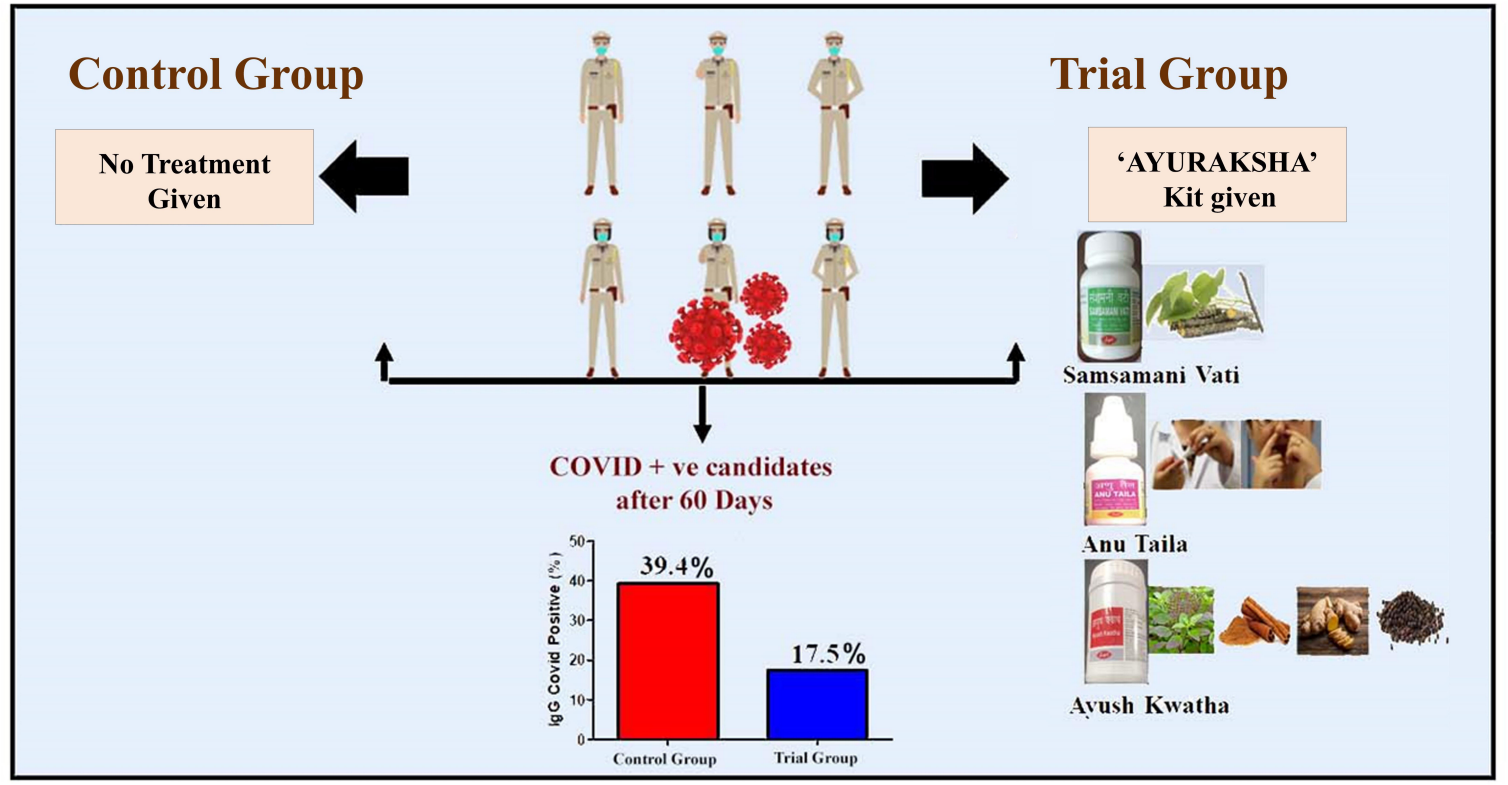
Summary of the study.

Further, the elevated glucose level is reported to enhance the viral replication with possible lethal complications via dysregulation of the immune system (74) while in our study, non-significant change in RBS level was seen in the trial group while a significant elevation was observed in the control group which indicates the potential role of Ayurveda-based prophylactic therapy in maintaining the blood sugar level of the candidate. A comparative analysis of baseline (before treatment) and after treatment data for the control and the trial group is shown in Supplementary Tables S7–S11 and the summary of the study has been depicted in Figure 3.

All the above observations have shown a significant role of AYURAKSHA Kit in reducing COVID-19 IgG positivity, improving the immunity and QoL of trial group during the Corona pandemic. This study explored the utilization of Ayurvedic traditional medicines for the prevention and management of such deadly diseases. Nevertheless, this study is a major milestone to serve the mankind in combating a COVID-19 outbreak and providing a valuable contribution toward the integration of Ayurvedic wealth into the modern science. The results also suggest the potential use of Indian Ayurvedic traditional herbal interventions as prophylaxis to prevent COVID-19.

### Limitations and future scope

Limitations of the study include non-uniform sample sizes of the study groups in which hematological investigations were done due to less availability of the control subjects. The study was not randomly allocated among the two groups due to the following reasons:

- During the first wave of COVID-19 outspread in Delhi, when all the law enforcement officers, including the police personnel, were at equal exposure and heightened risk of infection, non-randomization of subjects was done assuming the negligible probability of bias (if any).
- All the subjects (police personnel) were stuck due to their duty's obstacles, increased work consignments in different geographical locations of Delhi (to safeguard and maintain law and order) and travel-restricted facilities leading to their reduced availability and feasibility for randomization at the required point of time. Therefore, the convenient sampling was done.

Hence, bigger uniform randomized sample-sized hematological studies may be done in future. Inclusion of subjects with co-morbidities will be helpful in exploring the preventive percentage of AYURAKSHA kit in co-morbid population from the COVID-19 infection. Hence, AYURAKSHA kit may be used as a promising option for the management and prevention of COVID-19 infection as a stand-alone or integrative therapy globally.

## Conclusion

The world is still facing the COVID-19 pandemic, therefore, an integration of Ayurveda interventions with standards of care is the need of hour for the effective prevention and management of this infection. This study showed that about 55.6% protection was achieved against COVID-19 after 2 months of prophylactic intervention in trial group as compared to the control group, suggesting that AYURAKSHA kit if given, may prevent deterioration of COVID-19 disease into a more critical condition. The encouraging results will encourage the healthcare policy makers, stakeholders, and the researchers to the integration of both systems of medicines after in-depth research of AYURAKSHA kit (Ayurvedic immunity enhancers) for the prevention and control of the future deadly mutants' waves of COVID-19 pandemic (if any).

## Data availability statement

The raw data supporting the conclusions of this article will be made available by the authors, without undue reservation.

## Ethics statement

The studies involving human participants were reviewed and approved by the Institutional Ethics Committee of All India Institute of Ayurveda, New Delhi, India. The patients/participants provided their written informed consent to participate in this study.

## Author contributions

TN: conceptualization, study design, supervision, and critical review of the manuscript. SK: conceptualization, study design, and supervision. MV: data collection and supervision. VH, PP, MR, AM, SkR, SB, RY, VM, SM, RM, DK, RS, NB, PK, PD, MB, BB, AKM, GR, SG, JS, MD, and PP: data collection. SH: supplementary figures and data analysis. UT: supervision, intervention, and data collection. AV: review of manuscript. SR: data collection, laboratory investigations, interpretation, and review of manuscript. PY and AKa: intervention. AKu: data analysis, interpretation, and review of manuscript. HS: data cleaning, data interpretation, figures, writing, editing, and reviewing manuscript. RT: literature search, figures, data interpretation, writing of manuscript draft, editing, and reviewing. All authors contributed to the article and approved the submitted version.

## Funding

Funding was provided by the Ministry of AYUSH, India, for the medicine distribution and the data analysis. The funder of the study had no role in data collection, data analysis, data interpretation, or writing of the manuscript.

## Conflict of interest

The authors declare that the research was conducted in the absence of any commercial or financial relationships that could be construed as a potential conflict of interest.

## Publisher's note

All claims expressed in this article are solely those of the authors and do not necessarily represent those of their affiliated organizations, or those of the publisher, the editors and the reviewers. Any product that may be evaluated in this article, or claim that may be made by its manufacturer, is not guaranteed or endorsed by the publisher.

## Supplementary material

Supplementary Table S1 Socio-demographic characteristics of the participants in part-1 study participants.

Supplementary Table S2 Lifestyle characteristics of the participants in part-1 of the study.

Supplementary Table S3 Health-related characteristics.

Supplementary Table S4 Any health symptoms during past 12 months at the baseline and two follow-ups.

Supplementary Table S5 Compliance of the treatment.

Supplementary Table S6 Immunity and general health status at baseline and two follow-ups.

Supplementary Table S7 Blood profile of the trial and control groups.

Supplementary Table S8 Liver profile of the trial and control groups.

Supplementary Table S9 Cytokine profile of the trial and control groups.

Supplementary Table S10 Antibody levels in the trial and control groups.

Supplementary Table S11 Lymphocyte subset levels in the trial and control groups.

Supplementary Figure S1 Bar graph showing the feedback by Delhi Police Personnel depicting the usefulness of ‘AYURAKSHA' medicine.

Supplementary Figure S2 (a) Time Trend of COVID-19 Cases in Delhi. (b) Daily Incidence of COVID-19 cases in Delhi Police. COVID-19 Dashboard.

Supplementary Figure S3 (a) Status of COVID-19 in Delhi Police Personnel (Sept 21, 2020). (b) Age-specific (26–60 years) Mortality of Delhi Police vs. Delhi Population (As on 21st Sept 2020) In Percentage. (c) COVID 19 status of Police Personnel of different states.

## Abbreviations

ISQ: Immune Status Questionnaire
QoL: quality of life
ESR: erythrocyte sedimentation rate
CRP: C-reactive protein
IL: interleukin
RBS: random blood sugar
LDL: low density lipoprotein
CD: cluster of differentiation
BT: before treatment
AT: after treatment.
