# Supplementary material for: AYURAKSHA, a prophylactic Ayurvedic immunity boosting kit reducing positivity percentage of IgG COVID-19 among frontline Indian Delhi police personnel: A non-randomized controlled intervention trial
Source: Front Public Health. 2022 Aug 16;10:920126. doi: 10.3389/fpubh.2022.920126 (PMC9424736; doi:10.3389/fpubh.2022.920126)
Supplement: Supplementary Table S1 — Socio-demographic characteristics of the participants in part-1 study participants. [file Data_Sheet_4.docx]

**Table S1: Socio-demographic characteristics of the study in part-1 study participants**

| **Characteristics** | **N=47827** |
| --- | --- |
| **Mean age in years** (SD) (n=45199) | 40.9 (11.1) |
| **Age categories** (%) |  |
| ≤40 yrs | 22673 (50.2) |
| > 40 yrs | 22526 (49.8) |
| **Gender** (%) |  |
| Male | 42785 (89.3) |
| Female | 5102 (10.7) |
| **Religion** (%) (n=44039) |  |
| Hindu | 42817 (97.2) |
| Other religion | 1222 (2.8) |
| **Education** (%) (n=40917) |  |
| High School | 5720 (14.1) |
| Intermediate | 14481 (35.4) |
| Graduation | 18562 (45.4) |
| Post-Graduation | 2154 (5.3) |
| **Marital Status** (%) (n=38886) |  |
| Unmarried | 3318 (8.5) |
| Married | 35568 (91.5) |
| **Cadre** (%) (n=43819) |  |
| Constable | 19168 (43.7) |
| HC | 10479 (23.9) |
| Inspector | 4615 (10.5) |
| Other | 9557 (21.8) |

The socio-demographic characteristics of all study participants are illustrated in Table-S1. The mean of age of all participants was 40.9 years which were further categorised into two sub-categories- ≤ 40 years and > 40 years. 50.2% of participants were of ≤ 40 years of age and 49.8% were of > 40 years. Out of 47827 police personnel, the majority (89.3%) were males and belonged to Hindu community (97.2%). Education wise data suggested that among all participants, 5.3% were postgraduate, 45.4% were graduates, 35.4% twelfth, and 14.1% high school. The majority of the police personnel were married (91.5%). Among them, 43.7% were constable, 23.9% were headed constable and 10.5% were inspectors.

**Table S2: Lifestyle characteristics of the participants in part-1 of study**

| **Characteristics (N=47827)** | **N (%) / Mean (SD)** |
| --- | --- |
| **Food Habit N (%)** (N= 45282) | |
| Homemade **N (%)** | 36206 (80.0) |
| Outside food **N (%)** | 8515 (18.8) |
| Other **N (%)** | 561 (1.2) |
| Smoking **N (%)** | 1379 (2.9) |
| Chewing tobacco N (%) | 432 (0.9) |
| Alcohol drinking N (%) | 854 (1.8) |
| Yoga and meditation N (%) (n=45060) |  |
| Regular **N (%)** | 9515 (21.1) |
| Irregular **N (%)** | 31473 (69.9) |
| Never **N (%)** | 4072 (9.0) |
| Median day time sleep duration in hrs, Mean (SD) | 0 (0. 2) |
| Mean night time sleep duration in hrs, Mean (SD) | 7.3 (1.4) |

The lifestyle characteristics of all the study participants are described in Table-S2. Most of the police personnel were found to have the changed food habit during COVID, 80% (n=36206) of them were found to be homemade food consumers and only 18.8% were outside food consumers. It has been observed that only 2.9% of police personnel were smokers, 0.9% were having the habit of consuming smokeless tobacco, 1.8% were alcohol drinkers. 69.9% of participants had irregular yoga and meditation practice, however, (21.1%) were regular in yoga and meditation. 9% of them were those who never practiced yoga and Meditation. Sleep duration was not there at daytime and the mean ± SD of the sleep duration in hours at night was found to be 7.3 ± 1.4.

**Table S3: Health related characteristics**

| **Characteristics (N=47827)** | **N (%)** |
| --- | --- |
| Body Mass Index in m/kg2 (SD)(n=37426) | 24.9 (3.1) |
| Medical history of (past 3 months) |  |
| Diabetes (n=43008) | 1185 (2.8) |
| Hypertension (n=42981) | 1036 (2.4) |
| Any Allergy (past 3 months) |  |
| Cold (n=42861) | 513 (1.2) |
| Cough (n=42689) | 514 (1.2) |
| Breathing difficulty (n=42963) | 293 (0.7) |

The physical fitness and health related parameters were measured before treatment (BT) including body mass index (BMI), past three months medical history of diabetes, hypertension, allergies like cold, cough, and breathing difficulty (Table-S3). These measures showed that the mean ± SD of BMI of participants were 24.9 ± 3.1 which was normal, 1185 out of 43008 (2.8%) personnel were found to be diabetic, 1036 out of 42981 (2.4%) were hypertensive, 513 out of 42861 (1.2%) were having cold, 514 out of 42689 (1.2%) were having a cough, and 293 out of 42963 (0.7%) were facing breathing difficulties.

**Table S4: Any health symptoms during past 12 months at the baseline and two follow-ups**

| **Health Symptoms** | **Baseline**  **(day 0, n=47827)** | **First follow-up**  **(day 60, n=40446)** | **Second follow-up**  **(day-90, n=32806)** |
| --- | --- | --- | --- |
| **Cough (%)** | 77 (0.16) | 197 (0.53) (n=37327) | 60 (0.19)  (n=31448) |
| **Cold (%)** | 94 (0.20) | 202 (0.50)  (n=40441) | 54 (0.16)  (n=32802) |
| **Fever (%)** | 28 (0.06) | 134 (0.36)  (n=37388) | 50 (0.16)  (n=31526) |
| **Breathlessness (%)** | 9 (0.02) | 73 (0.20)  (n=37371) | 25 (0.08)  (n=31520) |
| **Tiredness (%)** | 3 (0.01) | 246 (0.66)  (n=37325) | 69 (0.22)  (n=31502) |
| **Body ache (%)** | 53 (0.11) | 207 (0.55)  (n=37328) | 57 (0.18)  (n=31511) |
| **Headache (%)** | 65 (0.14) | 239 (0.64)  (n=37324) | 60 (0.19)  (n=31490) |
| **Nasal Congestion (%)** | 0 | 120 (0.32)  (n=37321) | 24 (0.08)  (n=31425) |
| **Conjunctivitis (%)** | 0 | 59 (0.16)  (n=37315) | 18 (0.06)  (n=31368) |
| **Sore throat (%)** | 2 (0.00) | 85 (0.23)  (n=37307) | 20 (0.06)  (n=31340) |
| **Diarrhoea (%)** | 20 (0.04) | 44 (0.12)  (n=37328) | 12 (0.04)  (n=31356) |
| **Loss of taste (%)** | 0 | 55 (0.15)  (n=37330) | 20 (0.06)  (n=31368) |
| **Skin Rashes (%)** | 23 (0.05) | 45 (0.12)  (n=37318) | 27 (0.09)  (n=31364) |

Beside all above parameters, the table-S4 showed the details of health symptoms like cold, cough, fever, breathlessness, tiredness, body ache, headache, nasal congestion, conjunctivitis, sore throat, diarrhoea, loss of taste, skin rashes during past 12 months of all police personnel participants at the baseline BT (day-0) and at first (day 60) and second follow-up AT (day 90). It was observed that only 0.16% participants at baseline BT, 0.53% at first follow-up AT and only 0.19% participants at second follow-up AT had a cough history in past 12 months. Similarly, the cold and fever history of the past 12 months, was observed at baseline BT (0.20%; 0.06%), at first follow-up AT (0.50%, 0.36%), and second follow-up AT (0.16%, 0.16%) respectively. Breathlessness and Tiredness were observed in 0.02%; 0.01%participants at baseline BT, 0.20%; 0.66%participants at first follow-up AT and 0.08%, 0.22% at second follow-up AT respectively. Past history of symptoms like body ache, headache, nasal congestion, conjunctivitis was observed in 0.11%, 0.14%, 0.0 %, 0.0% participants at baseline BT; 0.55%, 0.64%,0.32%, 0.16% participants at first follow-up AT; and 0.18%, 0.19%, 0.08%, 0.06% at second follow-up AT respectively. Other histories of symptoms like sore throat, diarrhoea, loss of taste and skin rashes were noticed in 0.0%; 0.04%, 0.0%, 0.05 % participants at baseline BT, 0.23%; 0.12%, 0.15%, 0.12% participants at first follow-up AT and 0.06%, 0.04%, 0.06%, 0.09% at second follow-up AT respectively (Table-S4).

**Table S5: Compliance of the treatment**

| **Compliance** | **First follow-up (day 60)** | **Second follow-up (day 90)** |
| --- | --- | --- |
| **Tablets** | N=32737 | N=29132 |
| Regular, N (%) | 25616 (78.2) | 22545 (77.4) |
| Irregular, N (%) | 4484 (13.7) | 4644 (15.9) |
| Not taken, N (%) | 2637 (8.1) | 1943 (6.7) |
| **Kadha** | N=33044 | N=29394 |
| Regular, N (%) | 26625 (80.6) | 22129 (75.3) |
| Irregular, N (%) | 5067 (15.3) | 6290 (21.4) |
| Not taken, N (%) | 1352 (4.1) | 975 (3.3) |
| **Anu Taila application** | N=32626 | N=28956 |
| Regular, N (%) | 25397 (77.8) | 21809 (75.3) |
| Irregular, N (%) | 5131 (15.7) | 5722 (19.8) |
| Not taken, N (%) | 2098 (6.4) | 1425 (4.9) |
| **All three** | N=33238 | N=29546 |
| Regular for all 3, N (%) | 22854 (68.8) | 19273 (65.2) |
| Irregular for all 3, N (%) | 2402 (7.2) | 2891 (9.8) |
| Not taken at all, N (%) | 989 (3.0) | 615 (2.1) |
| Mixed, N (%) | 6993 (21.0) | 6767 (22.9) |

The detailed compliance of treatment during the first survey has been described after first (day 60) and second follow-up (day 90) AT in table-S5. In addition, First survey showed that the tablets of Sanshawani Vati (*T.cordifolia*)were consumed regularly by 78.2% participants during first follow-up AT and 77.4% participants during second follow-up AT. Similarly, AYUSH Kadha was regularly consumed by 80.6% during first follow-up AT and 75.3% participants during second follow-up AT. Alike above, Anu Taila (oil) was regularly applied by 77.8% participants during first follow-up AT and 75.3% participants during second follow-up AT (Table-S5). It was also observed that all three medicines [Samshamani Vati (*Tinospora cordifolia*) tablets, Kadha and Anu taila] were regularly used by 68.8% participants during first follow-up AT and 65.2% participants during second follow-up AT.

The second survey was done to find out the total percentage of compliance response rate of “AYURAKSHA” medicine and the results showed that a total of 60233 out of 67740 participants (89%) have taken “AYURAKSHA” medicine.

**Table S6: Immunity and General health status at baseline and two follow-ups**

*those with the immunity score of 6 or more

| **Immunity and Health** | **Baseline**  **(day 0, n=47827)** | **First follow-up**  **(day 60, n=40446)** | **Second follow-up**  **(day 90, n=32806)** |
| --- | --- | --- | --- |
| **Mean General Health score by participants (SD)** | 9.18 (1.36) (n=44393) | 9.27 (1.23) (n=38808) | 9.35 (1.20)  (n=31685) |
| **Immunity Status** | | | |
| **Mean (SD) ISQ score** | 9.53 (1.31) (n=45782) | 9.61 (1.19) (n=39880) | 9.54 (1.31) (n=32062) |
| **Good Immunity* (%)** | 44680 (97.59) | 39160 (98.19) | 31456 (98.11) |
| **Mean immune functioning score by participants (SD)** | 9.17 (1.32) (n=44226) | 9.26 (1.20)  (n=38718) | 9.34 (1.18) (n=31618) |
| **Reduced immune function by participants, N (%)** | 2281 (5.25)  (n=43440) | 1431 (3.76)  (n=38087) | 866 (2.74)  (n=31602) |

The detailed immunity and general health status of all participants at baseline (day 0), at first follow-up (day 60) after treatment (AT) and at second follow-up (day 90) after treatment (AT) has been depicted in table-S6. The data on the qualitative index of general health was measured on a scale of scores between 1 and 10. The data was presented on 2 points at 0 (bad general health) and 10 (good general health). It has been observed that there was no significant change in mean ± SD scores of participants at baseline BT (9.18 ± 1.36), after first follow-up (9.27 ± 1.23), and second follow-up (9.35 ± 1.20) AT scores respectively. The data on immune status was collected on 0–10-point scale using ISQ, where, 10 means good, 6 as normal and 0 as bad immunity. In table-4, good immunity status score (6 or more) was described. The data showed that there was no significant change observed in mean ± SD of ISQ score at all three levels, i.e., at baseline (9.53 ± 1.31), after first (9.61 ± 1.19), and second follow-up (9.54 ± 1.31) AT scores respectively. At baseline BT, 45782 participants showed good immunity (97.59%). However, the immunity percentage during the first follow-up (39160 [98.10%]) and second follow-up (31456[98.11]) AT was increased from the baseline data. The mean ± S.D score of immune functioning was found to be gradually improved from baseline BT (9.17 ± 1.32) to first (9.26 ± 1.20) and second (9.34 ± 1.18) follow-up AT, however, the difference was not significant. Hence, at baseline BT, 2281 participants out of 43440 (5.25%), at first follow-up AT, 1431 out of 38087 (3.76%) and at second follow-up AT 866 out of 31602 (2.74%) showed reduced or weakened immune function. This confirms that the percentage of a weakened immune system was found to be progressively decreased in participants from baseline BT (5.25%) to AT during first (3.76%) and second (2.74%) follow-up.

**Table S7: Table depicting the blood profile of Trial and Control Group**

|  | | | **Mean Levels** | | | | | | |
| --- | --- | --- | --- | --- | --- | --- | --- | --- | --- |
| **Sr. No.** |  | | **BT** | **AT** | **BT-AT** | **% Change** | **Std Err**  **(BT-AT)** | **t-test** | **p-value** |
| **1** | **HB** | Treated-N=80 | 14.33 | 14.73 | -0.4 | -2.8 | 0.07 | -5.00 | **.000*** |
|  |  | Control-N=66 | 14.35 | 14.64 | -0.29 | -2.02 | .06 | -4.75 | **.000*** |
| **2** | **TLC** | Treated-N=80 | 7.50 | 7.21 | 0.29 | 3.8 | .18 | 1.62 | .108 |
|  |  | Control-N=66 | 7.35 | 7.05 | 0.3 | 4.08 | .17 | 1.7 | .089 |
| **3** | **Neutrophils** | Treated-N=80 | 58.25 | 59.39 | -1.14 | -1.95 | .88 | -1.28 | .203 |
|  |  | Control-N=66 | 57.21 | 57.97 | -0.76 | -1.32 | 1.01 | -.75 | .455 |
| **4** | **Lymphocytes** | Treated-N=80 | 31.46 | 29.76 | 1.7 | 5.40 | .75 | 2.26 | **.027*** |
|  |  | Control-N=66 | 31.41 | 31.09 | 0.32 | 1.01 | .76 | .41 | .680 |
| **5** | **Monocytes** | Treated-N=80 | 7.358 | 7.76 | -0.4 | -5.46 | .65 | -.62 | .536 |
|  |  | Control-N=66 | 8.62 | 7.36 | 1.26 | 14.61 | .81 | 1.55 | .127 |
| **6** | **Eosinophils** | Treated-N=80 | 3.14 | 3.03 | 0.11 | 0.35 | .27 | .42 | .675 |
|  |  | Control-N=66 | 3.27 | 4.03 | -0.76 | -23.24 | .44 | -1.73 | .088 |
| **7** | **Basophils** | Treated-N=80 | 0 | 0 | 0 | 0 | 0 | 0 | 0 |
|  |  | Control-N=66 | .03 | .00 | 0.03 | 100 | .03 | 1.00 | .321 |
| **8** | **ESR** | Treated-N=80 | 17.91 | 15.31 | 2.6 | 14.51 | .68 | 3.83 | **.000*** |
|  |  | Control-N=66 | 19.35 | 14.68 | 4.67 | 24.13 | .77 | 6.09 | **.000*** |
| **9** | **PC** | Treated-N=80 | 178.15 | 193.35 | -15.2 | -8.53 | 4.51 | -3.37 | **.001*** |
|  |  | Control-N=66 | 189.58 | 195.88 | -6.3 | -3.32 | 5.36 | -1.18 | .244 |
| **10** | **CRP** | Treated-N=80 | 1.47 | 2.02 | -0.55 | 37.41 | .19 | -2.79 | **.007*** |
|  |  | Control-N=66 | 2.29 | 1.73 | 0.56 | 24.45 | .41 | 1.35 | .182 |

*p ≤ 0.05, considered as significant.

**Table S8: Table depicting the liver profile of Trial and Control group**

*p ≤ 0.05, considered as significant.

|  | | | **Mean Levels** | | | | | | |
| --- | --- | --- | --- | --- | --- | --- | --- | --- | --- |
| **Sr. No.** |  | | **BT** | **AT** | **BT-AT** | **% Change** | **Std Err**  **(BT-AT)** | **t-test** | **p-value** |
| **1** | **SGOT** | Treated-N=80 | 53.23 | 48.29 | 4.94 | 9.29 | 2.91 | 1.76 | .094 |
|  |  | Control-N=66 | 51.90 | 47.09 | 4.81 | 9.26 | 2.67 | 1.79 | .077 |
| **2** | **SGPT** | Treated-N=80 | 59.19 | 51.20 | 7.99 | 13.49 | 4.66 | 1.57 | .123 |
|  |  | Control-N=66 | 57.08 | 52.45 | 4.63 | 8.11 | 2.93 | 1.57 | .120 |
| **3** | **ALP** | Treated-N=80 | 102.44 | 99.30 | 3.14 | 3.06 | 3.05 | .49 | .308 |
|  |  | Control-N=66 | 99.47 | 97.65 | 1.82 | 1.82 | 3.69 | .49 | .623 |
| **4** | **Albumin** | Treated-N=80 | 3.66 | 3.63 | 0.03 | 0.81 | .06 | .47 | .642 |
|  |  | Control-N=66 | 3.91 | 3.55 | 0.36 | 9.20 | .074 | 4.84 | **.000*** |
| **5** | **Total Protein** | Treated-N=80 | 7.48 | 6.93 | 0.55 | 7.35 | .08 | 6.16 | **.000*** |
|  |  | Control-N=66 | 7.58 | 6.76 | 0.82 | 10.81 | .105 | 7.76 | **.000*** |
| **6** | **Bilirubin** | Treated-N=80 | 1.01 | .92 | 0.09 | 8.91 | .04 | 1.73 | .088 |
|  |  | Control-N=66 | .80 | .90 | -0.1 | -12.5 | .043 | -2.30 | **.025*** |
| **7** | **HDL** | Treated-N=80 | 56.53 | 45.32 | 11.21 | 19.83 | 1.88 | 5.96 | **.000*** |
|  |  | Control-N=66 | 58.75 | 46.97 | 11.78 | 20.05 | 1.94 | 6.07 | **.000*** |
| **8** | **LDL** | Treated-N=80 | 118.85 | 110.31 | 8.54 | 7.18 | 3.16 | 2.70 | **.008*** |
|  |  | Control-N=66 | 116.64 | 108.54 | 8.1 | 6.94 | 3.13 | 2.59 | **.012*** |

**Table S9 depicting the cytokine profile of Trial and Control Group**

|  | | | **Mean Levels** | | | | | | |
| --- | --- | --- | --- | --- | --- | --- | --- | --- | --- |
| **Sr. No.** |  | | **BT** | **AT** | **BT-AT** | **% Change** | **Std Err**  **(BT-AT)** | **t-test** | **p-value** |
| **1** | **IL6** | Treated-N=80 | 3.04 | 3.08 | -0.04 | -1.31 | .22 | -.17 | .865 |
|  |  | Control-N=66 | 2.73 | 3.42 | -0.62 | -25.27 | .30 | -2.25 | **.027*** |
| **2** | **IL2** | Treated-N=80 | .77 | .94 | -0.17 | -22.07 | .22 | -.74 | .461 |
|  |  | Control-N=66 | .60 | .42 | 0.2 | 33.33 | .17 | 1.00 | .317 |
| **3** | **IL4** | Treated-N=80 | 1.52 | 1.92 | -0.4 | -26.31 | .52 | -.76 | .449 |
|  |  | Control-N=66 | 2.01 | 1.80 | 0.21 | 10.44 | .48 | .43 | .665 |
| **4** | **IL10** | Treated-N=80 | 5.10 | 4.51 | 0.59 | 11.56 | .51 | 1.16 | .249 |
|  |  | Control-N=66 | 5.31 | 4.66 | 0.65 | 12.24 | .58 | 1.13 | .262 |
| **5** | **GI** | Treated-N=80 | 1.90 | 1.42 | 0.48 | 25.26 | .36 | 1.33 | .189 |
|  |  | Control-N=66 | 1.82 | 2.42 | -0.6 | -32.96 | .38 | -1.56 | .125 |
| **6** | **IL2** | Treated-N=80 | .63 | .84 | -0.21 | -33.33 | .13 | -1.54 | .128 |
|  |  | Control-N=66 | 10.16 | 9.13 | 1.03 | 10.13 | 1.35 | .76 | .447 |

*p ≤ 0.05, considered as significant.

**Table S10: Table depicting the Antibody levels in Trial and Control Group**

|  | | | **Mean Levels** | | | | | | |
| --- | --- | --- | --- | --- | --- | --- | --- | --- | --- |
| **Sr. No.** |  | | **BT** | **AT** | **BT-AT** | **% Change** | **Std Err**  **(BT-AT)** | **t-test** | **p-value** |
| **1** | **IgM** | Treated-N=80 | 4.13 | 2.86 | 1.27 | 30.75 | .41 | 3.03 | **.003*** |
|  |  | Control-N=66 | 3.33 | 3.33 | 0 | 0 | .45 | .08 | .994 |
| **2** | **IgG** | Treated-N=80 | 19.36 | 17.06 | 2.3 | 11.88 | .78 | 2.64 | **.010*** |
|  |  | Control-N=66 | 14.31 | 17.13 | -2.82 | -19.70 | .89 | -3.16 | **.002*** |
| **3** | **IgA** | Treated-N=80 | 3.36 | 3.14 | 0.22 | 6.54 | .16 | 1.35 | .181 |
|  |  | Control-N=66 | 2.67 | 3.19 | -0.52 | -19.47 | .17 | -3.02 | **.004*** |

*p ≤ 0.05, considered as significant.

|  | | | **Mean Levels** | | | | | | |
| --- | --- | --- | --- | --- | --- | --- | --- | --- | --- |
| **Sr. No.** |  | | BT | AT | BT-AT | % Change | Std Err  (BT-AT) | t-test | p-value |
| **1** | **CD3+** | Treated-N=80 | 1918.91 | 1649.66 | 269.25 | 14.03 | 63.45 | 4.24 | **.000*** |
|  |  | Control-N=66 | 2144.91 | 1693.56 | 451.35 | 21.04 | 111.84 | 4.03 | **.000*** |
| **2** | **Ratio**  **(CD3+/CD45)** | Treated-N=80 | 75.55 | 73.87 | 1.68 | 2.22 | .54 | 3.12 | **.003*** |
|  |  | Control-N=66 | 75.75 | 74.40 | 1.35 | 1.78 | 1.10 | 1.22 | .225 |
| **3** | **AbsolCD4+**  **T-helper cells** | Treated-N=80 | 1022.36 | 913.88 | 108.48 | 10.61 | 35.34 | 3.06 | **.003*** |
|  |  | Control-N=66 | 1141.91 | 909.58 | 232.33 | 20.34 | 60.32 | 3.85 | **.000*** |
| **4** | **Ratio**  **(CD3+/CD4+)** | Treated-N=80 | 40.31 | 41.42 | -1.11 | -2.75 | .62 | -1.77 | .080 |
|  |  | Control-N=66 | 40.93 | 40.76 | 0.17 | 0.41 | .65 | .25 | .801 |
| **5** | **Absolute CD8+** | Treated-N=80 | 797.66 | 668.79 | 128.87 | 16.15 | 37.28 | 3.45 | **.001*** |
|  |  | Control-N=66 | 866.33 | 679.7 | 186.64 | 21.54 | 50.28 | 3.71 | **0.000*** |
| **6** | **Ratio**  **(CD3+/CD4+)** | Treated-N=80 | 40.32 | 41.43 | -1.11 | -2.75 | 0.63 | 1.78 | 0.08 |
|  |  | Control-N=66 | 40.93 | 40.77 | 0.17 | 0.39 | 0.65 | 0.25 | 0.80 |
| **7** | **Ratio (CD4+/ CD8+)** | Treated-N=80 | 1.49 | 1.54 | -0.05 | -3.35 | 0.06 | 0.91 | 0.37 |
|  |  | Control-N=66 | 1.51 | 1.52 | -0.08 | -5.29 | 0.05 | 0.17 | 0.87 |

**Table S11: Table depicting the Lymphocyte subset levels in Trial and Control Group**

*p ≤ 0.05, considered as significant.
