# Supplementary figures and images for: AYURAKSHA, a prophylactic Ayurvedic immunity boosting kit reducing positivity percentage of IgG COVID-19 among frontline Indian Delhi police personnel: A non-randomized controlled intervention trial
Source: Front Public Health. 2022 Aug 16;10:920126. doi: 10.3389/fpubh.2022.920126 (PMC9424736; doi:10.3389/fpubh.2022.920126)

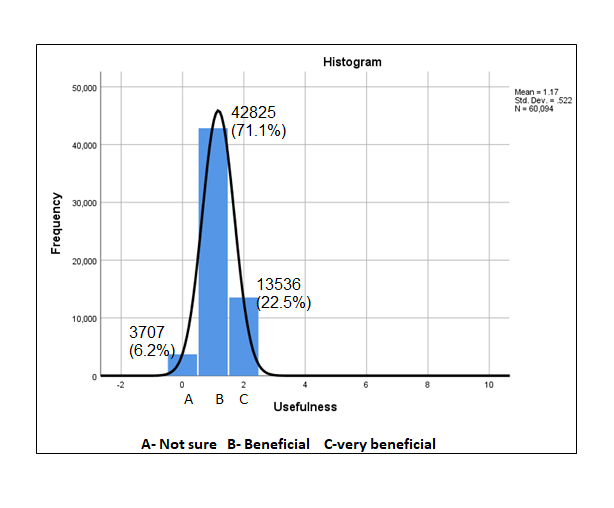

Supplement: Supplementary Figure S1 — Bar graph showing the feedback by Delhi Police Personnel depicting the usefulness of ‘AYURAKSHA' medicine. [file Image_1.JPEG]

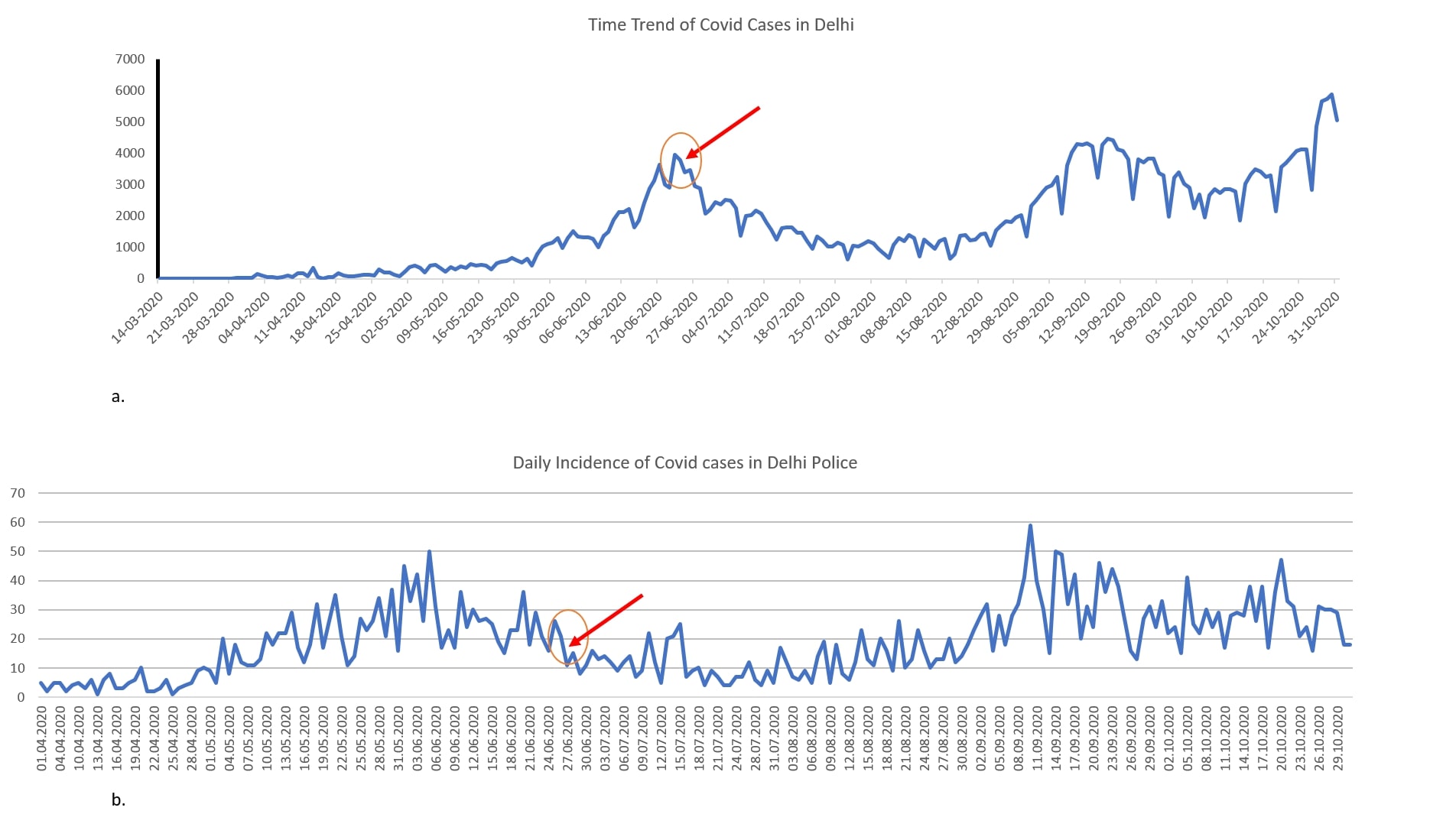

Supplement: Supplementary Figure S2 — (a) Time Trend of COVID-19 Cases in Delhi. (b) Daily Incidence of COVID-19 cases in Delhi Police. COVID-19 Dashboard. [file Image_2.JPEG]

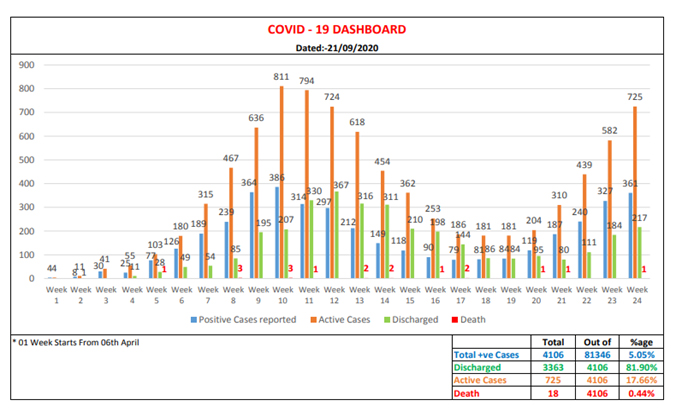

Supplement: Supplementary Figure S3 — (a) Status of COVID-19 in Delhi Police Personnel (Sept 21, 2020). (b) Age-specific (26–60 years) Mortality of Delhi Police vs. Delhi Population (As on 21st Sept 2020) In Percentage. (c) COVID 19 status of Police Personnel of different states. [file Image_3.JPEG]

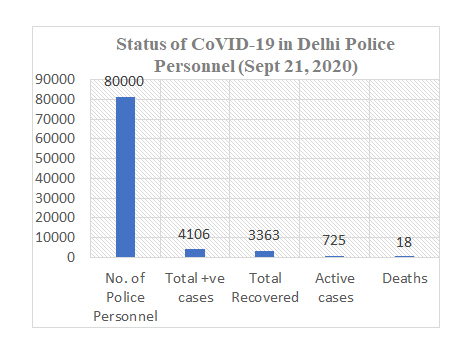

Supplement: Supplementary file 5 [file Image_4.JPEG]

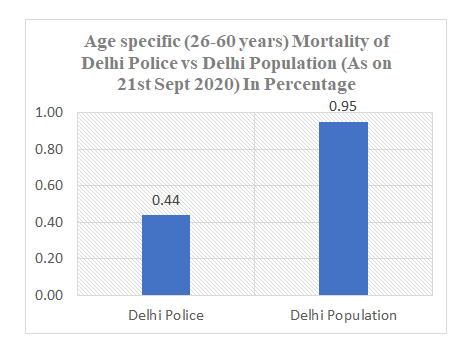

Supplement: Supplementary file 6 [file Image_5.JPEG]

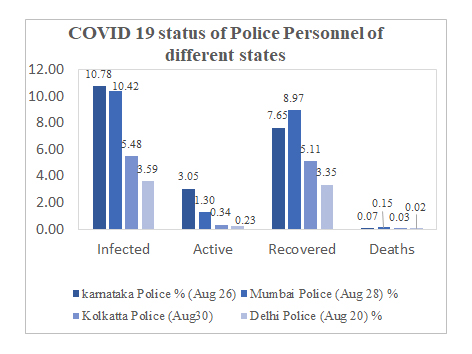

Supplement: Supplementary file 7 [file Image_6.JPEG]
