# Supplementary material for: AYURAKSHA, a prophylactic Ayurvedic immunity boosting kit reducing positivity percentage of IgG COVID-19 among frontline Indian Delhi police personnel: A non-randomized controlled intervention trial
Source: Front Public Health. 2022 Aug 16;10:920126. doi: 10.3389/fpubh.2022.920126 (PMC9424736; doi:10.3389/fpubh.2022.920126)
Supplement: Supplementary file 8 [file Data_Sheet_1.PDF]

CIN No.- U24233PB2008PTC032243

HERBHEAL
CARE BY NATURE

Herbal Health Research Consortium Pvt. Ltd.

(A GOVT. OF INDIA SPONSORED & PB. GOVT. ASSISTED AYUSH CLUSTER PROJECT)

Works at : Village Khyala Khurd, Ram Tirth Road, Amritsar
Ph.: 01858-262025, Fax.: 01858- 262026 E-mail: herbheal@ymail.com
Corrs. Add: 277, East Mohan Nagar, Amritsar - 143006

A Government Approved Testing Laboratory
Lic no. 1/ASU/TESTING LAB./PB./2014

Form No. 50 **Date: 08/05/2020**

CERTIFICATE OF ANALYSIS

(The Drugs and Cosmetics Act 1940 and the Rules Thereunder)

**Sample**	**AYUSH KWATHA CHOORNA**	**A.R. No.**	05/2020/TP/07
**Supplied by**	I.M.P.C.L	**Sample Quantity**	1*50g
**Mfd. by**	I.M.P.C.L[Lic. No. 19/Ay-Pb.(L.L)] HADBAST No. 334, V.P.O. NaagKalan-143601, Majitha Road, Via Majitha, Distt- Amritsar(Pb)	**Batch Size**	-
**Batch No.**	19-AKC-LDA-065	**D/M**	MAY-2020
**Date of receipt**	02/05/2020	**D/E**	APR-2022

Sr.No.	TESTS	RESULTS	SPECIFIC REQUIREMENTS
1.	**Description**	Complies	Brown color coarse powder with characteristic odor.
2.	**Loss On Drying (at 105°C)**	6.78%w/w	(Not more than 15.0% w/w)
3.	**Ash Value**	15.29%w/w	(Not more than 22.0% w/w)
4.	**Acid Insoluble Ash**	7.12%w/w	(Not more than 15.0% w/w)
5.	**Alcohol Soluble Extractive**	6.98%w/w	(Not less than 5.0%w/w)
6.	**Water Soluble Extractive**	9.32%w/w	(Not less than 5.0%w/w)
7.	**pH Value (10% aqueous solution)**	5.66	(4.5-6.0)
8.	**Microbial test**		
	a) Total Microbial Plate Count	170cfu/g	1 x 10^5 cfu/g
	b) Total Yeast and Moulds	98 cfu/g	1 x 10^3 cfu/g
9.	**Pathogen Tests**		
	a) *Escherichia coli*	Absent	Absent
	b) *Staphylococcus aureus*	Absent	Absent
	c) *Pseudomonas aeruginosa*	Absent	Absent
	d) *Salmonella*	Absent	Absent
10.	**Heavy Metals (By ICP-OES)**		
	a. Lead (as Pb)	Not Detected	NMT 10.0 ppm
	b. Cadmium (as Cd)	Not Detected	NMT 0.3 ppm
	c. Mercury (as Hg)	Not Detected	NMT 1.0 ppm
	d. Arsenic (as As)	Not Detected	NMT 3.0 ppm
11.	**Test For Aflatoxins (by TLC)** (B1, B2 and G1,G2)	Not Detected	B1 & G1- NMT 0.5 ppm<br>B2 & G2- NMT 0.1 ppm

Remarks: The Product complies with In-house specifications.

08/05/2020 08/05/2020 08/05/2020

Prepared By **Checked By** **Approved By**

"End of Report"

Note:

1. Sample(s) not drawn by us unless otherwise stated.
2. Total liability of this laboratory is limited to the invoiced amount.
3. Test certificate in full or parts shall not be used for promotional or publicity purpose.
4. The sample will be destroyed after one month of the date of issued test report, unless otherwise specified.

CIN No.- U24233PB2008PTC032243

HERBHEAL
CARE BY NATURE

Herbal Health Research Consortium Pvt. Ltd.

(A GOVT. OF INDIA SPONSORED & PB. GOVT. ASSISTED AYUSH CLUSTER PROJECT)
Works at : Village Khyala Khurd, Ram Tirth Road, Amritsar
Ph.: 01858-262025, Fax.: 01858- 262026 E-mail: herbheal@ymail.com
Corrs. Add: 277, East Mohan Nagar, Amritsar - 143006

A Government Approved Testing Laboratory

Form No. 50 | **Lic no. 1/ASU/TESTING LAB./PB./2014** | **Dated: 22/06/2020**

CERTIFICATE OF ANALYSIS

(The Drugs and Cosmetics Act 1940 and the Rules Thereunder)

**Sample**	**SANSAMANI VATI**	**A.R. No.**	06/2020/TP/126
**Supplied by**	I.M.P.C.L	**Sample Quantity**	1X100gm
**Mfd. by**	I.M.P.C.L[Lic. No. 19/Ay-Pb.(L.L)]<br>HADBAST No. 334, V.P.O. NaagKalan-143601,<br>Majitha Road, Via Majitha, Distt- Amritsar(Pb)	**Batch Size**	
**Batch No.**	19-AVG-LDA-414	**D/M**	JUNE-2020
**Date of receipt**	15/06/2020	**D/E**	MAY-2023

Sr.No.	TESTS	RESULTS	SPECIFIC REQUIREMENTS
1.	**Description**	Complies	A round biconvex, brown colour uncoated tablet with characteristic odour.
2.	**Loss On Drying (at 105°C)**	3.41% w/w	(Not more than8.00% w/w)
3.	**pH(5.0% Aqueous solution)**	6.41	(4.50-6.50)
4.	**Total Ash**	9.59% w/w	(Not more than15.00% w/w)
5.	**Acid Insoluble Ash**	11.68% w/w	(Not more than 15.00% w/w)
6.	**Alcohol Soluble Extractive**	11.84% w/w	(Not less than 7.00%w/w)
7.	**Water Soluble Extractive**	46.58% w/w	(Not less than40.00%w/w)
8.	**Average Weight**	252mg	(250mg±5%)
9.	**Disintegration Time**	22 Minutes	(Not more than 30 minutes)
10.	**Hardness**	4.2Kg/cm^2	(3.50-4.50Kg/cm^2)
11.	**Friability**	Complies	(Not more than 1.0% w/w)
12.	**Test For Aflatoxins (by TLC)**<br>(B1, B2 and G1,G2)	Not Detected	B1 & G1- NMT 0.5 ppm<br>B2 & G2- NMT 0.1 ppm
13.	**Microbial test**		
	a) Total Microbial Plate Count	281cfu/g	1 x 10^5 cfu/g
	b) Total Yeast and Moulds	32cfu/g	1 x 10^3 cfu/g
14.	**Pathogen Tests**		
	a) *Escherichia coli*	Absent	Absent
	b) *Staphylococcus aureus*	Absent	Absent
	c) *Pseudomonas aeruginosa*	Absent	Absent
	d) *Salmonella*	Absent	Absent
15.	**Heavy Metals (By ICP-OES)**		
	a) Lead (as Pb)	Not Detected	NMT 10.0 ppm
	b) Cadmium (as Cd)	Not Detected	NMT 0.3 ppm
	c) Mercury (as Hg)	Not Detected	NMT 1.0 ppm
	d) Arsenic (as As)	Not Detected	NMT 3.0 ppm

Remarks: The above submitted sample is of standard quality with respect of above tests.

Pooja 22/06/2020 — **Prepared By** | [illegible] 22/06/2020 — **Checked By** | Viney 22/06/2020 — **Approved By**

"End of Report"

Note:

1. Sample(s) not drawn by us unless otherwise stated.
2. Total liability of this laboratory is limited to the invoiced amount.
3. Test certificate in full or parts shall not be used for promotional or publicity purpose.
4. The sample will be destroyed after one month of the date of issued test report, unless otherwise specified.

CIN No.- U24233PB2008PTC032243

HERBHEAL
CARE BY NATURE

ISO 9001

Herbal Health Research Consortium Pvt. Ltd.

(A GOVT. OF INDIA SPONSORED & PB. GOVT. ASSISTED AYUSH CLUSTER PROJECT)
Works at : Village Khyala Khurd, Ram Tirth Road, Amritsar
Ph.: 01858-262025, Fax.: 01858- 262026 E-mail: herbheal@ymail.com
Corrs. Add: 277, East Mohan Nagar, Amritsar - 143006

Form No. 50

Government Approved Testing Laboratory
Lic no. 1/ASU/TESTING LAB./PB./2014

Dated: 09/05/2020

CERTIFICATE OF ANALYSIS

(The Drugs and Cosmetics Act 1940 and the Rules Thereunder)

Sample	ANU TAILA	A.R. No.	05/2020TP/021
Supplied by	I.M.P.C.L	Sample Quantity	1X 100 ml
Mfd. by	I.M.P.C.L [Mfg. Lic. No HP-43-Ay(L.L.)] Village: Kishanpura,P.O.- Gurumajra, Teh - Baddi, Distt: Solan (H.P.)	Batch Size	200 Litre
Batch No.	43-ATA-LDB-331	D/M	05/2020
Date of receipt	01/05/2020	D/E	04/2023

Sr.No.	TESTS	RESULTS	SPECIFICATIONS
1.	Description	Complies	A medicated oil with reddish brown colour.
2.	Odour	Complies	Characteristic
3.	Refractive index at 40°C	1.4657	1.4646-1.4659
4.	Specific gravity at 40°C	0.924	0.905-0.930
5.	Saponification value	196.55	188-200
6.	Iodine Value	98	88-106
7.	Acid Value	1.69	Not more than 3.00
8.	Peroxide Value	4.12	Not more than 6.00
9.	Mineral Oil	Absent	Absent
10.	Identification by TLC	Complies	Should be Complies
11.	Microbial test<br>a) Total Microbial Plate Count<br>b) Total Yeast and Moulds	 310 cfu/ml<br>40 cfu/ml	 1×10^5 cfu/ml<br>1×10^3 cfu/ml
12.	Pathogen Tests<br>a) *Escherichia coli*<br>b) *Staphylococcus aureus*<br>c) *Pseudomonas aeruginosa*<br>d) *Salmonella*	 Absent<br>Absent<br>Absent<br>Absent	 Absent<br>Absent<br>Absent<br>Absent
13.	Heavy Metals (By ICP-OES)<br>a) Lead (as Pb)<br>b) Cadmium (as Cd)<br>c) Mercury (as Hg)<br>d) Arsenic (as As)	 Not Detected<br>Not Detected<br>Not Detected<br>Not Detected	 NMT 10.0 ppm<br>NMT 0.3 ppm<br>NMT 1.0 ppm<br>NMT 3.0 ppm
14.	Test for Aflatoxins (By TLC)<br>(B1,B2 and G1,G2)	Not Detected	B1 & G1-NMT 0.5 ppm<br>B2 & G2-NMT 0.1 ppm

Remarks: The above submitted sample is of standard quality with respect of above tests.

Prepared By 09/05/2020 | Checked By 09/05/2020 | Approved By 09/05/2020

"End of report"

Note:

1. Sample(s) not drawn by us unless otherwise stated.
2. Total liability of this laboratory is limited to the invoiced amount.
3. Test certificate in full or parts shall not be used for promotional or publicity purpose.
4. The sample will be destroyed after one month of the date of issued test report, unless otherwise specified.
