# Supplementary material for: AYURAKSHA, a prophylactic Ayurvedic immunity boosting kit reducing positivity percentage of IgG COVID-19 among frontline Indian Delhi police personnel: A non-randomized controlled intervention trial
Source: Front Public Health. 2022 Aug 16;10:920126. doi: 10.3389/fpubh.2022.920126 (PMC9424736; doi:10.3389/fpubh.2022.920126)
Supplement: Supplementary file 9 [file Data_Sheet_2.PDF]

~~108~~ 103

अखिल भारतीय आयुर्वेद संस्थान

Ayuraksha

ALL INDIA INSTITUTE OF AYURVEDA (AIIA)

(आयुष मंत्रालय, भारत सरकार के अंतर्गत स्वायत्त संस्थान)

(An Autonomous Organization under the Ministry of AYUSH, Govt. of India)

GENERAL SCREENING PROFORMA - QUARANTINE

(To be filled by medical professional PG scholars)

Baseline (0 day)

1. DEMOGRAPHICS:

Registration No.: Date:

Name : Sex Male ☐ Female ☐

Address Age ____Years ___ Months

Ht (cms) - Weight (Kg) -

District: Zone:

Religion : Education : 10th/12th/Graduate/PG......

Marital Status : UM/ M / D / W Mobile number:

Date of quarantine started:

2. SIGNS AND SYMPTOMS ON ADMISSION:

SYMPTOMS			Mention the date of appearance of symptom	SYMPTOMS			Mention the date of appearance of symptom
2.1 History of fever	☐Yes	☐No		Abdominal pain	☐Yes	☐No	
2.2 Cough-							
2.2.1 dry	☐Yes	☐No		Headache	☐Yes	☐No	
2.2.2 with sputum production	☐Yes	☐No		Vomiting / Nausea	☐Yes	☐No	
				Diarrhoea	☐Yes	☐No	
2.3 Sore throat	☐Yes	☐No		Conjunctivitis	☐Yes	☐No	
2.4 Runny nose	☐Yes	☐No		Skin rash	☐Yes	☐No	
2.5 Wheezing	☐Yes	☐No		Skin ulcers	☐Yes	☐No	
2.6 Chest pain	☐Yes	☐No		Joint pain	☐Yes	☐No	
2.7 Muscle aches	☐Yes	☐No		Shortness of breath	☐Yes	☐No	
2.8 Fatigue	☐Yes	☐No		Inability to walk	☐Yes	☐No	
2.9 Other	☐Yes	☐No					

2.1 Any history of infection in family members: Yes ☐ No ☐, If yes who was affected –

Father ☐ Mother ☐ Brother ☐ Sister ☐ wife ☐ Husband ☐ Son ☐ Daughter ☐ Any other.....

2.2 History of Travel to abroad by self or any family member in last six months: Yes ☐ No ☐

3. Any Comorbidities:

1. Chronic cardiac disease *(not hypertension)*	Yes ☐No ☐Unk	7. CKD	☐Yes ☐No ☐Unk
2. Hypertension	☐Yes ☐No ☐Unk	8. CLD	☐Yes ☐No ☐Unk
3. Chronic lung disease	☐Yes ☐No ☐Unk	9. Chronic neurological disorder	☐Yes ☐No ☐Unk
4. Asthma	☐Yes ☐No ☐Unk	10. HIV	☐Yes ☐No ☐Unk
5. Diabetes	☐Yes ☐No ☐Unk	11. Tuberculosis	☐Yes ☐No ☐Unk
6. Any cancer	☐Yes ☐No ☐Unk	12. Any other:	

4. Type of food consumed: Homemade☐ Outside food ☐ Veg ☐ Non-Veg☐ Other ☐ ....

5. Addiction: Tobacco (Smoking ☐ Chewing ☐) Alcohol ☐ Drug abuse ☐ Other ☐ ....

6. Yoga & Meditation: Regular ☐ Irregularly ☐ Never ☐

7. Sleep: ______hrs. / day; ______hrs. / night

8. Hunger: Normal ☐ Medium ☐ Diminished ☐

9. Taste in mouth: Yes ☐ No☐

10. Digestive power: Normal ☐ Medium ☐ Diminished ☐

11. Bowel: Clear ☐, Mildly constipated ☐, Severe constipation ☐

Frequency: ______time / day, Odor:__________

Urine: Normal ☐, Less ☐, Excess ☐, Difficulty to pass ☐, Discolored ☐, Burning ☐

Frequency: ______times / day

104

12. Bala pariksha (strength assessment)

12.1 Assessment of Sharirika Prakriti (Body constitution)

Sl No	Particulars	VATA	PITTA	KAPHA
1	Your body constituency is	Lean	Moderate	Slight obese
2	Your complexion is	Dark and brown	Fair	Fair and bright.
3	Your skin is	Rough and Dry	Oily and hot	Oily and cold
4	Your veins are	More prominent	Less prominent	Not visible
5	Your eyes are	Small & darkish	Medium &brown	Big, attractive
6	Your nails are	Thin, Rough	Thin, Soft	Thick, Smooth
7	Your hair is	Rough, dry and less	Soft, oily and Premature graying	Black, thick and strong
8	Your Eye brows are	Thin, irregular	Thin	Thick and regular
9	Your forehead is	Narrow	Medium	Broad
10	Your appetite is	Variable and scanty	Good and excessive	Constant and less
11	Quantity of food	Variable	More	Less
12	Habit of taking food	Fast	Frequent	Slow
13	The food you like	Light, warm, sweet, sour and salt tastes	Cold, sweet, bitter and astringent taste	Hot, pungent, bitter and astringent taste
14	Your thirst is	Variable	Excessive	Scanty
15	You perspire (sweat)	Less	More	Foul smelling
16	Your sleep is	Scanty and Disturbed	Little but sound	Deep
17	Do you get tensed during difficulties	Always	Occasionally	Never
18	You start the work	Quickly	After thinking	After detailed thinking
19	Grasping power	Excellent	Moderate	Medium
20	Your memory	Poor	Excellent	Good
21	Your anger	For a short span	For more time	Less
22	You talk to the unknown persons	More	As per need	Less
23	You dream about	Running, flying, falling.	Shining objects like lightening, fire and violence	River, greenery, swimming.
24	Physical activity of your body is	Very active	Moderate As per need	Less active
25	You speak	Fast, talkative	Clear cut	Slow
26	Your mood	Changes quickly	Changes slowly	Unchangeable
27	Your resistance to disease	Poor	Average	Good
28	Competitive capacity	Don't like	Excellent competitor	Handle stress easily
29	Your financial status is	Poor, spends money quickly	Moderate, spends on luxuries	Rich, money saver, spends on food
30	Do you feel discomfort while working	In rainy season	In summer season	In winter season
	TOTAL			

PRAKRITI: Vata: ____ Pitta: _____ Kapha: _____

Dominant Dosha: Vata / Pitta/ Kapha **Recessive Dosha:** Vata / Pitta / Kapha

Prakriti: Vata Pitta / Vata Kapha / Pitta Kapha / Tridoshaja / Eka doshaja [Vata / Pitta / Kapha]

12.2 Deha bala: (Physical Strength)

Body strength - Can't do any type of work ☐, Ability to do partial work ☐, Energetic enough ☐

12.3 Dhatu sara pareeksha:

- ***Rasa/Twak sara***: Soft, oily and glowing skin ☐, Sparse and silky hair on the skin ☐
- ***Rakta sara***: Oily and reddish palpebral conjunctiva ☐, Reddish lounge, nail, palm ☐
- ***Mamsa sara***: Stable ☐,well-built and bulky ☐, Prominent Neck muscles , UL & LL ☐
- ***Meda sara:*** Oily lips, hair, nails ☐, Good voice ☐
- ***Asthi sara:*** Prominent joint, chin and collar bone ☐
- ***Majja sara:*** Oily complexion☐ Having soft voice☐, Having firm,round,long and prominent joints☐
- ***Shukra sara:*** Milky white and lustrous eyes ☐, Pleasing personality ☐, heavy built in the region of the hip ☐, Well build body ☐

12.4 Satmya: (Adaptability)

-Change in food habits - Most of the edibles are suitable ☐, Few edibles are suitable ☐, Very few edibles are suitable ☐.

-Change in place - never troublesome ☐, sometimes troublesome ☐, always troublesome ☐

-Change in season - never troublesome ☐, sometimes troublesome ☐, always troublesome ☐

12.5 Chetasa bala: (Mental Strength)

-Feeling of well-being - Not feeling well physically and mentally ☐, Moderately feels well ☐, Completely feels well ☐

-Dreams: Daily 1 or 2 ☐, Frequently (twice a week) ☐, Occasionally (once a week)☐, No dreams ☐

-Functioning of mind, intellect and sensoria - Loss of concentration and enthusiasm ☐, Less enthusiastic and not interested in any work ☐, Normal enthusiasm and vigor ☐

-Fears: Not at all (never experienced) ☐-0, Sometimes experienced ☐-1, Frequently experienced but the daily activities are not affected ☐-2,Fears most of the time and it affects routine activities ☐-3

-Weakness: Does not feel weakness in routine activities ☐-0, Sometime feels weakness in routine activities ☐-1, Most of the time feels weakness in routine activities ☐-2, Bed ridden and feels excessive weakness ☐-3

-Worries: Never worries ☐-0, Worries some time ☐-1, Worries even in performing simple acts ☐-2, Most of the time worried ☐

13. Laboratory investigations: (to be obtained from the medical staff at quarantine center)

1	TC		☐	9	Neutrophils		☐
2	Monocytes		☐	10	Lymphocytes		☐
3	Basophils		☐	11	Eosinophils		☐
4	Monocytes		☐	12	ESR		☐
5	CD 4		☐	13	CD 8		☐
6	IL 6		☐	14	B lymphocytes		☐
7	T lymphocyte		☐	15	Th1, Th2 cells		☐
8	Natural killer cells		☐	16	Other		☐
			☐				☐
			☐				☐
			☐				☐

14.

Please indicate how often you have had the following complaints in the past 12 months:

	Never	Sometimes	Regularly	Often	(Almost) always
Sudden high fever					
Diarrhea					
Headache					
Skin problems (e.g. acne & eczema)					
Muscle and joint pain					
Common Cold					
Coughing					

14.1 **How you feel at this moment?**

A	I score my general health the following grade (0 = very bad; 10 = very good)	....	....
B	I score my immune functioning the following grade (0 = very bad; 10 = very good)	....	....
C	Do you have reduced immune functioning at this moment?	Yes	No
D	Do you have a chronic disease? If yes, please specify:	Yes	No

14.2 ISQ Scoring instructions.

Raw Score	Final Score
≥15	0
14	1
13	2
11, 12	3
10	4
8, 9	5
7	6
6	7
5	8
3, 4	9
≤2	10

Each item of the ISQ can be scored as follows: Never = 0 points; Sometimes = 1 point; Regularly = 2 points; Often = 3 points; (Almost) always = 4 points; Calculate the sum score of the 7 ISQ items. To obtain the final ISQ score, translate the "raw" ISQ scores as follows: Interpretation: 0 = very poor, 10 excellent perceived immune status. Cut off for reduced immune functioning: ISQ < 6.

15. WHOQOL- BES

		Very Poor	Poor	Neither poor nor good	Good	Very good
1 (G1)	How would you rate your quality of life?	1	2	3	4	5
		Very dissatisfied	Dissatisfied	Neither satisfied nor dissatisfied	Satisfied	Very satisfied
2 (G4)	How satisfied are you with your health	1	2	3	4	5

15.1 The following questions ask about how much you have experienced certain thins n he last two weeks

		Not at all	A little	A moderate amount	Very much	An extreme amount
3 (F1.4)	To what extent do you feel that physical pain prevents you from doing what you need to do?	1	2	3	4	5
4(F11.3)	How much do you need any medical treatment to function in your daily life?	1	2	3	4	5
5(F4.1)	How much do you enjoy life?	1	2	3	4	5
6(F24.2)	To what extent do you feel your life to be meaningful?	1	2	3	4	5
		Not at all	A little	A moderate amount	Very much	Extremely
7(F5.3)	How well are you able to concentrate?	1	2	3	4	5
8 (F16.1)	How safe do you feel in your daily life	1	2	3	4	5
9 (F22.1)	How healthy is your physical environment?	1	2	3	4	5

15.2 The following questions ask about how completely you experience or were able to do certain things in the last two weeks.

		Not at all	A little	Moderately	Mostly	Completely
10 (F2.1)	Do you have enough energy for everyday life?	1	2	3	4	5
11 (F7.1)	Are you able to accept your bodily appearance?	1	2	3	4	5
12 (F18.1)	Have you enough money to meet your needs?	1	2	3	4	5
13 (F20.1)	How available to you is the information that you need in your day-to-day life?	1	2	3	4	5
14 (F21.1)	To what extent do you have the opportunity for leisure activities?	1	2	3	4	5
		Very Poor	Poor	Neither poor no good	Good	Very good
15 (F9.1)	How well are you able to get around?	1	2	3	4	5

92

108

15.3 The following questions ask you to say how **good or satisfied** you have felt about various aspects of your life over the last two weeks.

		Very dissatisfied	Dissatisfied	Neither satisfied nor dissatisfied	Satisfied	Very satisfied
16 (F3.3)	How satisfied are you with your sleep?	1	2	3	4	5
17 (F10.3)	How satisfied are you with your ability to perform your daily living activities?	1	2	3	4	5
18(F12.4)	How satisfied are you with your capacity for work?	1	2	3	4	5
19 (F6.3)	How satisfied are you with yourself?	1	2	3	4	5
20(F13.3)	How satisfied are you with your personal relationships?	1	2	3	4	5
21(F15.3)	How satisfied are you with your sex life?	1	2	3	4	5
22(F14.4)	How satisfied are you with the support you get from your friends	1	2	3	4	5
23(F17.3)	How satisfied are you with the conditions of your living place?	1	2	3	4	5
24(F19.3)	How satisfied are you with your access to health services?	1	2	3	4	5
25(F23.3)	How satisfied are you with your transport?	1	2	3	4	5
		Never	Seldom	Quite often	Very often	Always
26 (F8.1)	How often do you have negative feelings such as blue mood, despair, anxiety, depression?	1	2	3	4	5

Did someone help you to fill out this form?..

How long did it take to fill this form out?..

Do you have any comments about the assessment?

16. Date to come on for next assessment ______________________

17. Date of medicine started:

1. Name and signature of PG scholar:

2. Name and sign of Nodal officer:

3. Name of coordinator:
