# Supplementary material for: AYURAKSHA, a prophylactic Ayurvedic immunity boosting kit reducing positivity percentage of IgG COVID-19 among frontline Indian Delhi police personnel: A non-randomized controlled intervention trial
Source: Front Public Health. 2022 Aug 16;10:920126. doi: 10.3389/fpubh.2022.920126 (PMC9424736; doi:10.3389/fpubh.2022.920126)
Supplement: Supplementary file 10 [file Data_Sheet_3.PDF]

Comparative analysis of incidence and mortality of COVID-19 of Delhi police and the General Delhi population

The comparative analysis of incidence and mortality of COVID-19 of Delhi police and the General Delhi population was done. The data were taken from the dashboard data of Delhi police personnel and general population data were obtained and confirmed from the following sources:

1. "*Coronavirus Outbreak in India" - https://www Covid19india.org.;*
2. *"https://www.mohfw.gov.in/"*
3. *"https://www.thehindu.com/news/national/india-coronavirus-lockdown-june-25-2020-live-updates/article31911410.ece"*

In addition, the incidence of COVID-19 among Delhi police was also compared with the police personnel of three other states-Karnataka, Kolkatta, and Mumbai whose data were obtained from the data source: (As Assessed on 31/08/2020)

1. *https://timesofindia.indiatimes.com/city/mumbai/as-5-more-die-maharashtra-police-toll-150-this-month-worst-with-47-fatalities/articleshow/77814877.cms*
2. *https://www.newindianexpress.com/states/karnataka/2019/jul/20/fill-16838-police-posts-by-end-of-2019-says-karnataka-high-court-2006716.html*
3. *https://timesofindia.indiatimes.com/city/bengaluru/most-cops-who-died-of-*Covid-*were-on-field-duty-75-aged-above-50/articleshow/77749458.cms*
4. *https://m.timesofindia.com/city/kolkata/50-drop-in-new-*Covid-*cases-in-kolkata-police/amp_articleshow/77827390.cms*

*https://www.deccanherald.com/national/east-and-northeast/kolkata-police-acp-dies-of-*Covid-*19-876100.html*
